# Eco-friendly synthesis of silver oxide nanoparticles using *Nepeta cataria* L. (Lamiaceae) flowers extract: a multifaceted study of their antimicrobial and hemocompatible potential

**DOI:** 10.1038/s41598-026-53571-8

**Published:** 2026-05-21

**Authors:** Shahira A. Hassoubah, Shaza Yehya Qattan, Zeina Walid Sharawi, Alawiah Mohammad Saleh Alhebshi, Azhar Abdullah Najjar, Hansa Gul, Muhammad Nauman Khan, Nouman Ahmad, Tewekel Melese Gemechu

**Affiliations:** 1 Department of Biological Sciences, Faculty of Science, King Abdulaziz University, P.O. Box 80203, Jeddah, 21589 Saudi Arabia; 2 Department of Zoology, University of Mianwali, Mianwali, Punjab Pakistan; 3 Department of Botany, University of Chakwal, Chakwal, Punjab Pakistan; 4 Department of Biotechnology, University of Mianwali, Mianwali, Punjab Pakistan; 5 Department of Natural Resources Management, Ambo University, P.O. Box 19, Ambo, Ethiopia

**Keywords:** *N. cataria* L. flowers, Characterization, Antibacterial assay, Hemocompatible activities, Biochemistry, Biological techniques, Biotechnology, Chemistry, Drug discovery, Microbiology, Nanoscience and technology

## Abstract

This is the first report on the green synthesis of silver oxide nanoparticles (Ag_2_ONPs) using *Nepeta cataria* flower extract as a novel bioreducing and capping agent in an aqueous medium. This study aimed to describe these distinctive nanoparticles and explore their versatile biomedical applications through in silico molecular docking. UV-Vis, FTIR, XRD, SEM-EDX, Zeta Potential and DLS were used to characterize the synthesized Ag_2_ONPs. Their biological effects were measured based on antibacterial, antifungal, hemolytic, anticoagulant, and thrombolytic activities. Characterization confirmed the crystallization and formation of stable, crystalline, quasi-spherical Ag_2_ONPs, with an average crystallite size of 20.7 nm (XRD) and a particle size of 39 ± 2.4 nm (SEM). The zeta potential − 39 mV indicated excellent colloidal stability. The Ag_2_ONPs exhibited strong antibacterial activity (maximum inhibition zone of 30 mm against *Bacillus subtilis*) and potent antifungal activity (up to 83 mm) against *Trichophyton rubrum*. These nanoparticles were highly hemocompatible and exhibited strong anticoagulant and thrombolytic properties. To provide deeper insight into their antimicrobial action, this study uniquely integrated in silico molecular docking to explore interactions between the nanoparticles and key microbial protein targets. In summary, *N. cataria* L. flowers represent a promising new source of biocompatible Ag_2_ONPs with potent antimicrobial and hemo-modulatory properties, making them potential candidates for future therapeutic applications.

**Supplementary Information:**

The online version contains supplementary material available at 10.1038/s41598-026-53571-8.

## Introduction

In the rapidly evolving field of nanoscience, manipulating matter at atomic and molecular scales has unlocked materials with previously unattainable properties. Metallic nanoparticles (NPs) are miniature powerhouses reshaping industries from medicine to manufacturing^1^. Silver oxide nanoparticles (Ag_2_ONPs), in particular, have become a focal point of research, celebrated for their exceptional surface-to-volume ratio and distinctive optical behavior, which grant them remarkable antimicrobial and multifunctional potential^2^. They are used for targeted drug delivery in cancer therapy^3^, as high-contrast agents in bioimaging^4^, to prevent bacterial growth in dental materials^5^, and to support tissue regeneration during wound healing^6^. Recent studies have also emphasized their dual capability to cure infections and enhance cell proliferation efficiency in various cell lines^7^. In environmental and industrial applications, they are used for disinfection of pathogens in water treatment^8^, catalysis^9^, biosensors to detect pollutants^10^, and antiviral coatings^11^. In addition, Ag_2_ONPs have been used in agriculture to safeguard crops^12^, to enhance the efficiency of renewable energy systems^13^, and in food packaging to increase shelf life^14^.

Traditional methods for synthesizing Ag_2_ONPs include chemical processes, such as reduction reactions, and physical processes, such as evaporation-condensation and laser ablation. The use of these techniques has been associated with several issues, including high cost, significant time and energy consumption, labor intensity, and toxicity. This requires the development of new methods for the safe production of Ag_2_ONPs. In recent years, green synthesis approaches have been used to fabricate nanoparticles that are environmentally friendly, low-cost, non-toxic, and exhibit enhanced activities. Algae, bacteria^15^, fungi, and plant extracts, among other biomaterials, are used as reducing agents in the green synthesis of nanoparticles. Plant extracts, in particular, offer several advantages over other biological methods owing to their ubiquitous availability, minimal management requirements, biosafety, and affordability. Various plant extracts have been effectively applied in this respect; for example, leaf extracts of *Azadirachta indica* A. Juss. (neem) have formed AgNPs with antimicrobial and wound healing properties^16^, whereas leaf extracts of *Capparis zeylanica* L. have demonstrated potent antimicrobial and antiproliferative efficiency^17^. Additionally, extracts of *Carica papaya* L. (papaya)^18^ and *Ocimum sanctum* L. (tulsi)^19^ have produced nanoparticles with strong antibacterial activity. Similarly, AgNPs with strong antimicrobial and antioxidant properties have been synthesized using rhizome extracts from plants such as *Curcuma longa* L. (turmeric)^20^ and *Zingiber officinale* Roscoe (ginger)^21^. The botanical method is flexible and is also used to purify water with extracts from the fruit peel of *Citrus limon* (L.) Osbeck (lemon)^22^, to detect pollutants like hydrogen peroxide using leaf extracts of *Atalantia monophylla* (L.) DC. ^23^, to use and leaf extracts of *Moringa oleifera* Lam. for anticancer and water treatment applications^24^.

Building on this green synthesis framework, the present study utilized an extract from a plant known for its rich medicinal profile. We selected *N. cataria*, a species in the Lamiaceae family, which is well known for its abundance of bioactive secondary metabolites. With a history of use dating back to ancient times, *N. cataria* L. has been documented to possess a wide spectrum of pharmacological activities, including anticancer, antimicrobial, and anti-inflammatory effects^25^. The significance of *N. cataria* L. in nanotechnology is further underscored by recent research demonstrating the successful application of AgNPs for non-toxic sterilization and germination of its seeds, highlighting a synergistic and safe relationship between this plant and silver nanomaterials^26^. Although various parts of the plant are known to be active, this study focuses on a novel approach using its flower extract as a bioreducing and capping agent. We report the first successful synthesis of Ag_2_ONPs using its flowers and conduct a comprehensive investigation of their biological potential. Our study specifically investigated the antifungal and antibacterial activities of the synthesized Ag_2_ONPs, highlighting their multifaceted therapeutic potential.

## Materials and methods

### Plant sample collection and identification

Flowers of *N. cataria* were collected from Susoom Valley, Chitral, Pakistan, on privately owned land belonging to one of the authors, and identified by Dr. Muhammad Nauman Khan, Department of Botany, Islamia College Peshawar, Peshawar, Pakistan. A voucher specimen was deposited in the public herbarium of the Department of Botany, Islamia College Peshawar, Peshawar, Pakistan, under voucher/specimen number Nauman Bot. 00290. (ICP). This species is not protected and is not listed as endangered. Therefore, no specific permissions or licenses were required for collection. All procedures complied with relevant institutional, national, and international guidelines and legislation. All chemicals used in this study were of analytical grade and purchased from commercial suppliers. Silver nitrate (AgNO₃, ≥ 99.9%, Merck), sodium hydroxide (NaOH, ≥ 98%, Merck), and hydrochloric acid (HCl, 37%, Merck) were used for pH adjustment when necessary. Potassium bromide (KBr, ≥ 99%, Merck) was used for pellet formation during FTIR analyses. For antimicrobial assessment, Mueller-Hinton agar and tryptic soy agar (Merck) media were prepared, and the antibacterial effect was determined against the representative Gram-positive bacteria *Staphylococcus aureus* (ATCC 25923) and *Bacillus subtilis* (ATCC 19659), as well as the Gram-negative bacterium, *Escherichia coli* (ATCC 25922). Antifungal activity was tested against clinically relevant fungi, including *Candida albicans* (ATCC 10231), *Aspergillus niger* (ATCC 16404), *Cryptococcus neoformans* (ATCC 208821), and *Trichophyton rubrum* (ATCC 28188). All solutions were freshly prepared using distilled water. The microbial strains were obtained from the Biochemistry Laboratory, Institute of Chemistry, University of Sargodha, Sargodha, Pakistan.

### Preparation of plant extract

Freshly collected *N. cataria* L. flowers were washed with distilled water to remove dust and surface impurities, then shade-dried for 20 days at an ambient temperature of approximately 25 °C. The dried sample was ground into a fine powder and sieved through US standard mesh No. 70. Approximately 25 g of the powdered sample was mixed with 500 mL of distilled water in a volumetric flask and allowed to stand at room temperature for 5 h. The mixture was then stirred vigorously on a hot plate for 3 h to ensure complete extraction of the analytes. The extract was filtered using Whatman No. 42 filter paper, and the filtrate was placed in clean airtight containers for use in further experiments.

### **Synthesis of silver oxide NPs (**Ag_2_ONPs)

A 10 mL sample of *N. cataria* L. flower extract (NCFE) was mixed with 10 mL of a 1.0 mM aqueous silver nitrate solution (AgNO_3_) to synthesize green Ag_2_ONPs. The flower extract of *N. cataria* L. was used as both a reducing agent and a stabilizing agent in the synthesis of Ag_2_ONPs. The reaction mixture was exposed to direct sunlight for 25 min to initiate the photochemical reduction of silver ions. The formation of NPs was observed visually by the gradual color change of the solution from colorless to dark brown due to surface plasmon resonance, indicating the formation of Ag_2_ONPs. The colloidal suspension was centrifuged at 10,000 rpm for 30 min to isolate the NPs. To remove any unreacted ions and free phytochemicals, the pellet was washed several times with distilled water and centrifuged under the same conditions. Some of the dried nanoparticles were reserved for physicochemical characterization, while the remaining Ag_2_ONPs were redispersed into a uniform and stable colloidal suspension by bath sonication in distilled water. The resulting nanoparticles were designated as Ag_2_ONPs, and further studies were conducted using these nanoparticles. All sunlight-assisted synthesis experiments were performed on clear, sunny days between 10:00 AM and 2:00 PM under direct solar exposure (UV to IR spectrum) following the methodology of ^27^, with modifications. The UV index was monitored using a mobile application and maintained between 7 and 10 to ensure consistent irradiation conditions. No drug loading or encapsulation was performed in this study, as the work focused solely on the green synthesis and biological evaluation of pure Ag_2_ONPs.

### Characterization of synthesized Ag_2_ONPs

The biosynthesized Ag_2_ONPs were characterized for their morphology, stability, particle size distribution, surface functional groups, and crystalline nature using different analytical techniques. The reduction of silver ions was observed by a color change of the reaction mixture to brown, confirming the initial formation of Ag_2_ONPs. Optical characteristics were examined by measuring the UV-visible absorption spectrum at 300–800 nm, using a Cecil Aquarius CE 7200 dual-beam spectrophotometer. The phase purity and crystalline structure were verified by X-ray diffraction (XRD) with a JDX-3532 diffractometer (JEOL, Tokyo, Japan). The capping and stabilization of Ag₂ONPs by phytochemical functional groups present in the plant extract were analyzed using Fourier-transform infrared (FTIR) spectroscopy (IRSpirit-T, Shimadzu, Japan) over a spectral range of 400–4000 cm⁻¹. For analysis, the dried nanoparticle powder was thoroughly mixed with potassium bromide (KBr), finely ground, and compressed into a transparent pellet using the KBr pellet technique prior to measurement^28^ Surface morphology and elemental analysis of the biosynthesized Ag_2_ONPs was analyzed by SEM and EDX (JEOL JSM-5910). The hydrodynamic size and surface charge of the NPs were analyzed by DLS (Malvern Zetasizer Nano ZS). A simultaneous TGA/DSC analyzer (SDT 650, TA Instruments, USA) was used to determine the thermal stability of Ag_2_ONPs. The measurements were conducted under nitrogen from room temperature to 900 °C at a heating rate of 10 °C min^− 1^.

### Antibacterial assay

The antibacterial activity of Ag_2_ONPs was evaluated against key human pathogenic bacteria, including representative Gram-positive strains (*S. aureus* ATCC 25923 and *B. subtilis* (ATCC 19659), and the Gram-negative strain *E. coli* (ATCC 25922). The test was conducted using the agar well diffusion method following a standard protocol^29^, with slight modifications to optimize experimental conditions.

For the antibacterial assay, Mueller-Hinton (MH) agar was prepared by dissolving 5.3 g of agar powder in 125 mL of deionized water. The medium was autoclaved at 121 °C and 15 psi for 20 min. After sterilization, the Molten agar was cooled to approximately 50 °C. Then, 25 mL of the medium was poured into sterile Petri plates and allowed to solidify. The standardized bacterial suspension was evenly spread on the agar surface using a sterile cotton swab.

The agar was aseptically marked with four wells, each 6 mm in diameter, using a sterile cork borer. To determine antibacterial activity, several test solutions were added to the wells. A higher concentration (30 µg/mL) of biosynthesized Ag_2_ONPs was added to well (a) to assess the inherent antibacterial property of the plant extract. A standard antibiotic solution (Gentamicin, 30 µg/mL) was added to well (b) as a positive control. A lower concentration (15 µg/mL) of biosynthesized Ag_2_ONPs was added to well (c), and an aqueous extract of *N. cataria* (30 µg/mL) was added to well (d).

The plates were incubated at 37 °C for 24 h, and the antibacterial activity of each test sample after incubation was tested by measuring the diameter of the clear zone of inhibition (ZOI) around each well in millimeters (mm). Each experiment was conducted three times, and the findings are expressed as the mean ± standard deviation (SD) to achieve reliability and statistical precision. The Minimum Inhibitory Concentration (MIC) of the biosynthesized Ag_2_ONPs was determined using the broth microdilution method. Serial dilutions of Ag_2_ONPs were prepared in MH broth at concentrations ranging from 5 to 100 µg/mL. For each dilution, 100 µL of a standardized bacterial inoculum (approximately 1.5 × 10^8^ CFU/mL) was added. The culture tubes were incubated at 37 °C with continuous agitation to enhance mixing and ensure exposure of the bacteria to the NPs. A tube containing only the bacterial suspension served as the positive control, and a sterile broth served as the negative control. The MIC was determined as the lowest concentration of Ag_2_ONPs that showed no visible bacterial growth, indicating a bacteriostatic effect.

### Antifungal assay

The antifungal potential of green-synthesized silver oxide nanoparticles (Ag₂ONPs) derived from *N. cataria* flower extract was tested against four clinically relevant pathogenic fungi: *C. albicans* (ATCC 10231), *A. niger* (ATCC 16404), *C. neoformans* (ATCC 208821), and *Trichophyton rubrum* (ATCC 28188), following the protocol of^27^, with changes. All fungal isolates were grown on Sabouraud Dextrose Agar (SDA) plates and incubated at 28 °C. The inocula were prepared as follows: yeast cells of *C. albicans* and *C. neoformans* and spores of *A. niger* and *T. rubrum*, were removed from freshly grown cultures and suspended in sterile normal saline. The turbidity of each suspension was adjusted to approximately 15 × 10^6^ CFU/mL according to the McFarland standard, ensuring a consistent inoculum density. For antifungal analysis, 5 mL of Sabouraud Dextrose Broth (SDB) was transferred to sterile test tubes and spiked with biosynthesized Ag_2_ONPs to a final concentration of 30 µg/mL. Sterile distilled water was used to ensure that the total volume of all samples was consistent. A 100 µL aliquot of the standardized fungal suspension was inoculated into each tube. Terbinafine (30 µg/mL) served as a positive control, whereas uninoculated broth and inoculated broth without nanoparticles were used as negative controls. The tubes were placed in a slanted position to improve aeration and interaction with the surface, and then incubated at 28 °C for 48 h. The growth in the nanoparticle-treated tubes was visually compared with the controls to assess antifungal efficacy.

### Hemolytic potential assay

To determine the biocompatibility of the produced Ag_2_ONPs with red blood cells (RBCs), an in vitro hemolysis assay was performed. The process was based on the method described by Das and Saikia et al. ^30^, with minor modifications. Fresh human blood was collected from one of the authors, who served as a healthy volunteer donor, after obtaining informed consent. The blood was collected in an anticoagulant vial containing sodium heparin and was used immediately for the hemolytic potential assay. The blood was centrifuged at 3000 rpm for 10 min and the plasma was discarded. The remaining RBC pellet was washed three times with chilled phosphate-buffered saline (PBS, pH 7.4) to obtain a pure RBC suspension. In the assay, the washed RBC suspension was incubated with different concentrations of Ag_2_ONPs (0.5–3.5 µg/mL). Triton X-100 served as a positive control to induce 100% hemolysis, while PBS was used as a negative control. All samples were incubated at 37 °C after light mixing. Following incubation, the tubes were centrifuged, and the degree of hemolysis was determined by measuring the absorbance of the released hemoglobin at 540 nm using a UV-Vis spectrophotometer. The percentage of hemolysis was determined using the following formula:

$$\:\begin{array}{cccc}&\:\%Hemolysis=\frac{\mathrm{A}\mathrm{b}\mathrm{s}\mathrm{o}\mathrm{r}\mathrm{b}\mathrm{a}\mathrm{n}\mathrm{c}\mathrm{e}\:\mathrm{o}\mathrm{f}\:\mathrm{S}\mathrm{a}\mathrm{m}\mathrm{p}\mathrm{l}\mathrm{e}\:-\:\mathrm{A}\mathrm{b}\mathrm{s}\mathrm{o}\mathrm{r}\mathrm{b}\mathrm{a}\mathrm{n}\mathrm{c}\mathrm{e}\:\mathrm{o}\mathrm{f}\:\mathrm{N}\mathrm{e}\mathrm{g}\mathrm{a}\mathrm{t}\mathrm{i}\mathrm{v}\mathrm{e}\:\mathrm{C}\mathrm{o}\mathrm{n}\mathrm{t}\mathrm{r}\mathrm{o}\mathrm{l})}{\mathrm{A}\mathrm{b}\mathrm{s}\mathrm{o}\mathrm{r}\mathrm{b}\mathrm{a}\mathrm{n}\mathrm{c}\mathrm{e}\:\mathrm{o}\mathrm{f}\:\mathrm{P}\mathrm{o}\mathrm{s}\mathrm{i}\mathrm{t}\mathrm{i}\mathrm{v}\mathrm{e}\:\mathrm{C}\mathrm{o}\mathrm{n}\mathrm{t}\mathrm{r}\mathrm{o}\mathrm{l}\:-\:\mathrm{A}\mathrm{b}\mathrm{s}\mathrm{o}\mathrm{r}\mathrm{b}\mathrm{a}\mathrm{n}\mathrm{c}\mathrm{e}\:\mathrm{o}\mathrm{f}\:\mathrm{N}\mathrm{e}\mathrm{g}\mathrm{a}\mathrm{t}\mathrm{i}\mathrm{v}\mathrm{e}\:\mathrm{C}\mathrm{o}\mathrm{n}\mathrm{t}\mathrm{r}\mathrm{o}\mathrm{l}}&\:&\:\mathrm{x}\mathrm{100}\end{array}$$

### Anticoagulant activity

Prevention of blood clotting by Ag_2_ONPs was evaluated using a modified version of the protocol outlined by Ajarem et al. ^31^. The same freshly collected human blood sample, obtained from a healthy author-donor with informed consent, was used for the anticoagulant activity assay. The blood was aliquoted (1 mL per tube) into five test tubes. Ag_2_ONPs were synthesized and added to four tubes to attain concentrations of 10, 20, 30, and 40 µg/mL. The fifth tube, containing only untreated blood, served as a negative control. The tubes were incubated at 37 °C and observed after 1 h to assess blood clot formation and determine the anticoagulant effect of the Ag_2_ONPs.

### Thrombolytic activity

The ability of the produced Ag_2_ONPs to dissolve existing blood clots was determined following the process of Azeez et al. ^32^. Fresh human blood collected from the same healthy author-donor with informed consent was used to prepare the clots for the thrombolytic activity assay. A fresh drop of human blood was placed on a glass slide and allowed to clot for 20 min. Five samples were used (A-E) including a negative control (A). Various concentrations of Ag_2_ONPs (10, 20, 30, and 40 µg/mL) were applied to the formed clots. The slides were incubated for 1 h, and time-lapse observation was performed to visually assess the extent of clot lysis and dissolution, as evidence of the thrombolytic potential of the nanoparticles.

### Molecular docking

#### Protein selection and preparation

In this study, several target proteins from different pathogenic microorganisms were selected to investigate their potential interactions with silver oxide nanoparticles (Ag₂ONPs). The selected proteins represent crucial enzymatic and structural components involved in the virulence, metabolism, and survival of these pathogens. From *C. neoformans*, the protein Laccase-1 (AlphaFold ID: AF-Q55P57-F1-v6) was selected because it plays a significant role in melanin production and fungal pathogenicity^33^. Similarly, the Hsp90-like protein from *T. rubrum* (AlphaFold ID: AF-F2SVB4-F1-v6) was included because of its involvement in the stress response and fungal growth regulation^34^. The FDC1 protein from *(A) niger* (PDB ID: 4ZA7)^35^ and the Aspartic Proteinase (SAP2 gene product) from *C. albicans* (PDB ID: 1EAG) were selected for their key roles in tissue invasion and virulence^36^. Additionally, the DNA gyrase subunit B from *S. aureus* (PDB ID: 3U2D)^37^, the FtsZ protein (PDB ID: 2VAM), which is essential for DNA replication and survival of *(B) subtilis*^38^ and FimH from *E. coli* K-12 (PDB ID: 4XO8) were also targeted due to their critical functions in bacterial adhesion and biofilm formation^39^. Collectively, these proteins were chosen as molecular targets to evaluate the potential antimicrobial mechanism of AgNPs using in silico interaction and docking studies.

The wizard in the Schrödinger Suite 2025-1 was used to prepare the 3D structures of the selected proteins. The preparation procedure included removing unnecessary water molecules, optimizing bond orders, and inserting any missing side-chain or loop atoms with the Prime module. The protein structures were then minimized to constrained energy minimization of using Optimized Potentials for Liquid Simulations-3 (OPLS3) force field, and the root-mean-square deviation (RMSD) of the crystallographic heavy atoms was minimized until it converged to 0.3 Å ^40^.

#### Ligand preparation

As a representative structure of the ligand, the PubChem database was searched using PubChem CID: 92,152 to identify, silver oxide (AgO)^41^, as a representative structure of the synthesized Ag_2_O (Ag_2_ONPs). The structure of the AgO ligand was minimized using the LigPrep module of the Schrodinger Suite 2025-1 through desalting and geometric optimization. The Epik module was used to prepare the ionization and tautomeric forms of the ligand within the physiological pH range of 7.0 ± 0.2. Finally, an OPLS force field was used to minimize the ligand. Docking analysis was performed on the resulting optimized ligand conformation^40^.

#### Molecular docking protocol

The Schrodinger Suite 2025-1 Glide module was used for molecular docking^42^. For every protein, a receptor grid was created to identify the active site, with the centroid of the binding pocket defined by the coordinates of the cocrystallized ligand in the original PDB structure. The AgO ligand was docked to the active site (Table 1). The resulting binding poses were analyzed and ranked using the Glide g-score (G-Score). This scoring term estimates binding affinity by considering important molecular interactions, rewarding favorable lipophilic, hydrogen bonding, and metal-ligand interactions, and penalizing steric clashes. The binding mode with the most favorable (i.e. lowest) docking score for each protein-ligand complex was selected for further analysis.

To further quantify the thermodynamic stability of the protein-nanoparticle complexes and evaluate the individual energy contributions governing binding, Prime MM-GBSA (Molecular Mechanics-Generalized Born Surface Area) calculations were performed in the Schrodinger Suite 2025-1. This procedure enabled estimation of the total binding free energy (ΔG_bind_) and its decomposition into individual energetic components, such as van der Waals interactions (ΔG_vdW_), hydrogen bonding contributions (ΔG_hbond_), Coulombic/ electrostatic interactions (ΔG_coulomb_), and generalized Born solvation energy ((ΔG_solv_GB_). This decomposition offers physicochemical insight into the hydrophobic and polar forces that stabilize the complexes and underlie their antimicrobial action.

**Table 1:** Grid generation parameters for molecular docking.

Receptor ID	Grid Center (X, Y, Z)	Inner Box (Å)	Outer Box (Å)
4XO8	-20.708, -5.722, -15.573	10 × 10 × 10	24.791 × 24.791 × 24.791
3U2D	0.432, 2.710, 24.385	10 × 10 × 10	26.817 × 26.817 × 26.817
2VAM	38.325, -1.698, 7.795	10 × 10 × 10	23.170 × 23.170 × 23.170
AF-Q55P57-F1-v6	-10.186, -7.834, -14.582	10 × 10 × 10	23.883 × 23.883 × 23.883
AF-F2SVB4-F1-v6	-19.185, -15.047, 24.440	10 × 10 × 10	24.013 × 24.013 × 24.013
1EAG	41.455, 24.800, 13.156	10 × 10 × 10	28.919 × 28.919 × 28.919
4ZA7	22.119, 9.901, 23.363	10 × 10 × 10	26.428 × 26.428 × 26.428

### Statistical evaluation

All experiments were performed in triplicate (*n* = 3) to ensure the reliability and reproducibility of the results. Data are expressed as mean ± standard deviation (SD). Statistical analyses and graphical representations were performed using Statistix version 8.1 (Analytical Software, USA), Microsoft Office 2016 Plus, and OriginPro 2021. One-way analysis of variance (ANOVA) with Tukey’s honestly significant difference (HSD) post-hoc test was used to compare the differences between treatments. Statistical significance was set at *P* ≤ 0.05.

## Results and discussion

### UV-visible spectroscopy (UV) analysis

**Fig. 1:**
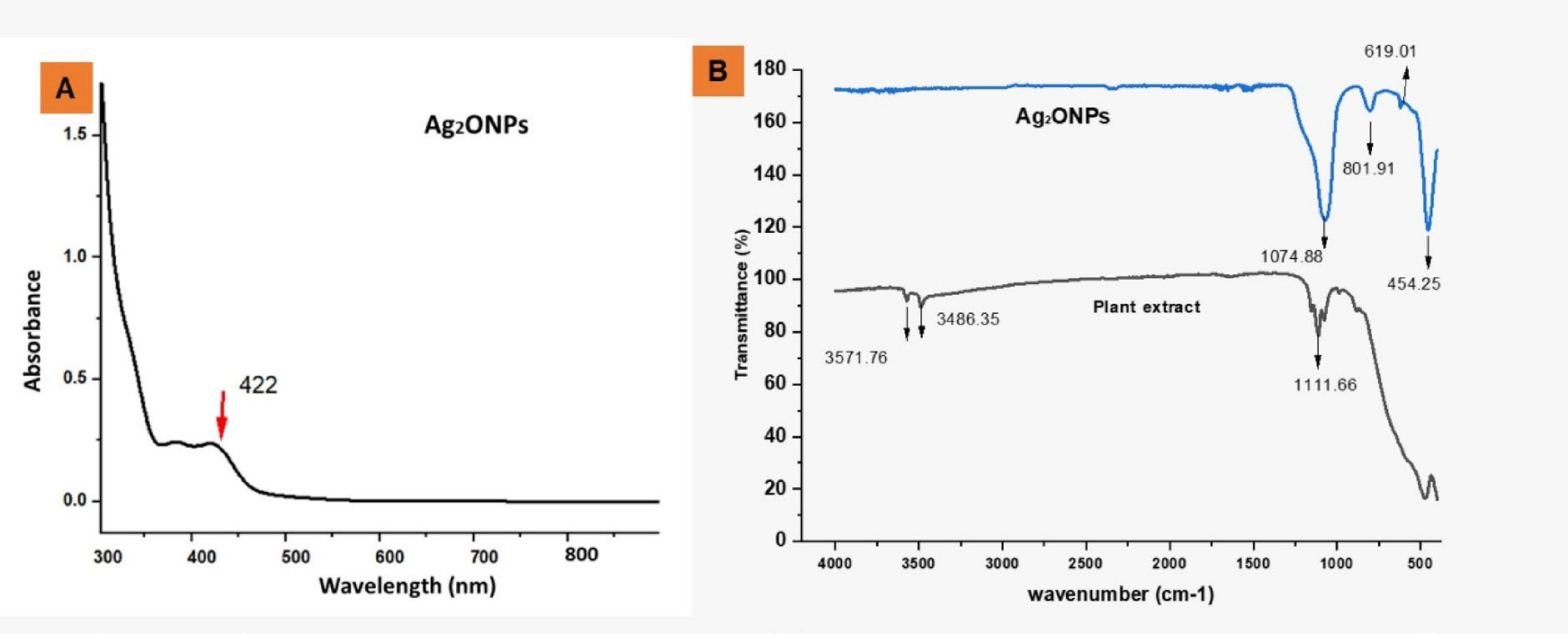
(**A**) Uv-Spectroscopy of Ag_2_ONPs (**B**) FTIR of Ag_2_ONPs.

The successful green synthesis of Ag₂ONPs was confirmed by both visual and spectroscopic analyses, as shown in Fig. 1A. When *N. cataria* L. flower extract was added to the aqueous silver nitrate solution, a rapid color change to a stable reddish-brown hue was observed, indicating the bioreduction of silver ions. This formation was further validated by UV-visible spectroscopy, which revealed a distinct and sharp Surface Plasmon Resonance (SPR) peak centered at 422 nm. The presence of this characteristic absorption band confirms the synthesis of Ag_2_ONPs and suggests that the nanoparticles are predominantly spherical, small in size, and well-dispersed within the colloidal solution, without significant aggregation^43^.

### FTIR analysis to identify functional groups

Fourier-Transform Infrared (FTIR) spectroscopy was employed to identify the functional groups present in the *N. cataria* L. flower extract and to confirm their role in the reduction, capping, and stabilization of the Ag_2_ONPs. The FTIR spectra for both the pure plant extract and Ag_2_ONPs are presented in Fig. 1B.

Several characteristic absorption bands were observed in the spectrum of the *N. cataria* L. plant extract (black line), reflecting the complex phytochemical composition of the extract. A large broad band appears in the interval 3571 –3486 cm^− 1^ which can be assigned to the O-H stretching vibrations of hydroxyl groups in polyphenols, flavonoids, and alcohols^44^, as well as possible N-H stretching of amines or amides in the extract. These hydroxyl groups are essential because they are the main moieties responsible for the reduction of Ag ions. Additionally maximum absorption was observed at 1111 cm^− 1^ and 1074 cm^− 1^. These bands are associated with the C-O stretching vibrations of alcohol ethers and carboxylic acids, which are widespread functional groups in secondary metabolites of medicinal plants^45,46^.

When comparing the FTIR spectrum of the biosynthesized Ag_2_ONPs (blue line), significant changes are evident, indicating the successful formation of nanoparticles as well as the involvement of plant biomolecules in the process. Notably, the wide O-H band observed in the plant extract is greatly reduced in the Ag_2_ONPs spectrum. This decrease indicates that the hydroxyl groups of the plant’s phytochemicals were oxidized during the redox process of the silver ions and are now involved in capping the nanoparticle surface. This observation is supported by the phytochemical profile reported by Nadeem et al. (2022), which revealed the existence of many hydroxyl-rich compounds in *N. cataria* L. extracts, including phenols, phytol, and terpenoids such as endo-Borneol. It is probable that these molecules are the major bio-reducing and stabilizing molecules in our synthesis^25^.

More importantly, the Ag_2_ONPs spectrum displays several new and sharp peaks in the lower wavenumber regions. The strong absorption warrants at 801.91 cm^− 1^, 619.01 cm^− 1^ and 454.25 cm^− 1^ are typical of metal-oxygen (M-O) bonds^47,48^. These peaks can be specifically assigned to the Ag-O stretching and bending vibrational modes, providing definitive evidence for the successful synthesis of silver oxide. The peak at approximately 619 cm^− 1^ is a well-documented characteristic stretching vibration of the Ag-O bond in the Ag_2_ONPs lattice structure^49,50^.

In conclusion, FTIR analysis confirmed that the bioactive compounds (likely polyphenols and flavonoids) rich in hydroxyl and carbonyl groups, in the *N. cataria* L. flower extract served as effective bio-reducing and stabilizing agents. The disappearance of key functional group peaks from the extract and the appearance of strong, characteristic Ag-O vibrational bands in the NPs spectrum verify that these biomolecules successfully capped the nanoparticles and that the final product was Ag₂ONPs.

###  X-ray diffraction (XRD) **Analysis**

XRD analysis confirmed the successful synthesis of crystalline, metallic Ag_2_ONPs. The diffraction pattern exhibited four characteristic peaks at 2θ values of ~ 32°, ~ 38°, ~ 55°, and ~ 65.3°, corresponding to the (111), (200), (311), and (400) planes of the face-centered cubic (FCC) structure of Ag_2_O, respectively. These results are in excellent agreement with (JCPDS Card No. 41-1104 ^51^. Similar results were reported in ^52,53^. The absence of diffraction peaks corresponding to other silver oxide phases, particularly the characteristic Ag_2_ONPs peak at 32.8°, confirms that the synthesized product is silver oxide. Notably, the diffraction peak at 38.1°, assigned to the (200) plane of Ag_2_O, is in a region where peaks for metallic silver (Ag°) can also appear. As discussed in the literature, the close overlap between the Ag and Ag_2_ONPs diffraction peaks around this angle can indicate the formation of a hybrid structure^52^. In particular, Ullah et al. ^54^, noted that the peak at approximately 38° could be assigned to both metallic Ag and Ag_2_ONPs, confirming the simultaneous existence of both phases in their samples(38.1^0^, JCPDS card No. 65-2871, 04-0783 and 38.0^o^, JCPDS card No. 41-1104)^52^. Therefore, while the dominant phase in our synthesized product is clearly crystalline Ag_2_ONPs, the presence of the (200) peak suggests the formation of a composite material rather than a purely metallic silver phase. This confirms the successful green synthesis of stable, crystalline Ag_2_ONPs.

The relatively stronger intensity of the (111) reflection compared to other peaks suggests preferential growth along this plane, which can be attributed to the selective adsorption of plant-derived phytochemicals on specific crystallographic faces during nucleation and growth processes.

The average crystallite size of the Ag_2_ONPs was determined using the Debye–Scherrer Eqs.^55,56^:

1 $$D = \frac{{K\lambda }}{{\beta \cos \theta }}$$

where *K* is the shape factor (0.9), *λ* is the X-ray wavelength of the Cu Kα radiation (0.15406 nm), *β* is the full width at half maximum (FWHM) of the most intense diffraction peak in radians, and *θ* is the Bragg angle. The (111) peak at 2θ = 32.7° was used for the calculations, with *β* = 0.40° (0.00698 rad) and *θ* = 16.35°. Substituting these values yields:

2 $$D = \frac{{0.9 \times 0.15406}}{{0.00698 \times \cos \left( {16.35^\circ } \right)}} = 20.7~nm$$

confirming the nanometer-scale dimension of the particles.

The lattice parameter (*a*) of the cubic Ag₂O crystal was determined using Bragg’s law and the cubic lattice relation.

3 $$d = \frac{\lambda }{{2\sin \theta }}$$

4 $$a = d\sqrt {h^{2} + k^{2} + l^{2} }$$

For the (111) plane, the interplanar spacing was *d* = 0.2736 nm, and the corresponding lattice parameter was calculated as

5 $$a = 0.2736 \times \sqrt 3 = 0.474~nm$$

This value aligns well with the standard lattice constant for bulk Ag₂O (0.4736 nm), as supported by Pham et al. ^51^, further verifying the formation of the pure FCC phase.

To gain deeper insight into the structural characteristics, the microstrain (ε), dislocation density (δ), and degree of crystallinity were evaluated using the following equations:

6 $$\varepsilon = \frac{\beta }{{4\tan \theta }}$$

7 $$\delta = \frac{1}{{D^{2} }}$$

8 $$Degree~of~Crystallinity = \frac{{Area~of~Crystalline~Peaks}}{{Total~Area~under~the~Curve}} \times 100$$

**Fig. 2:**
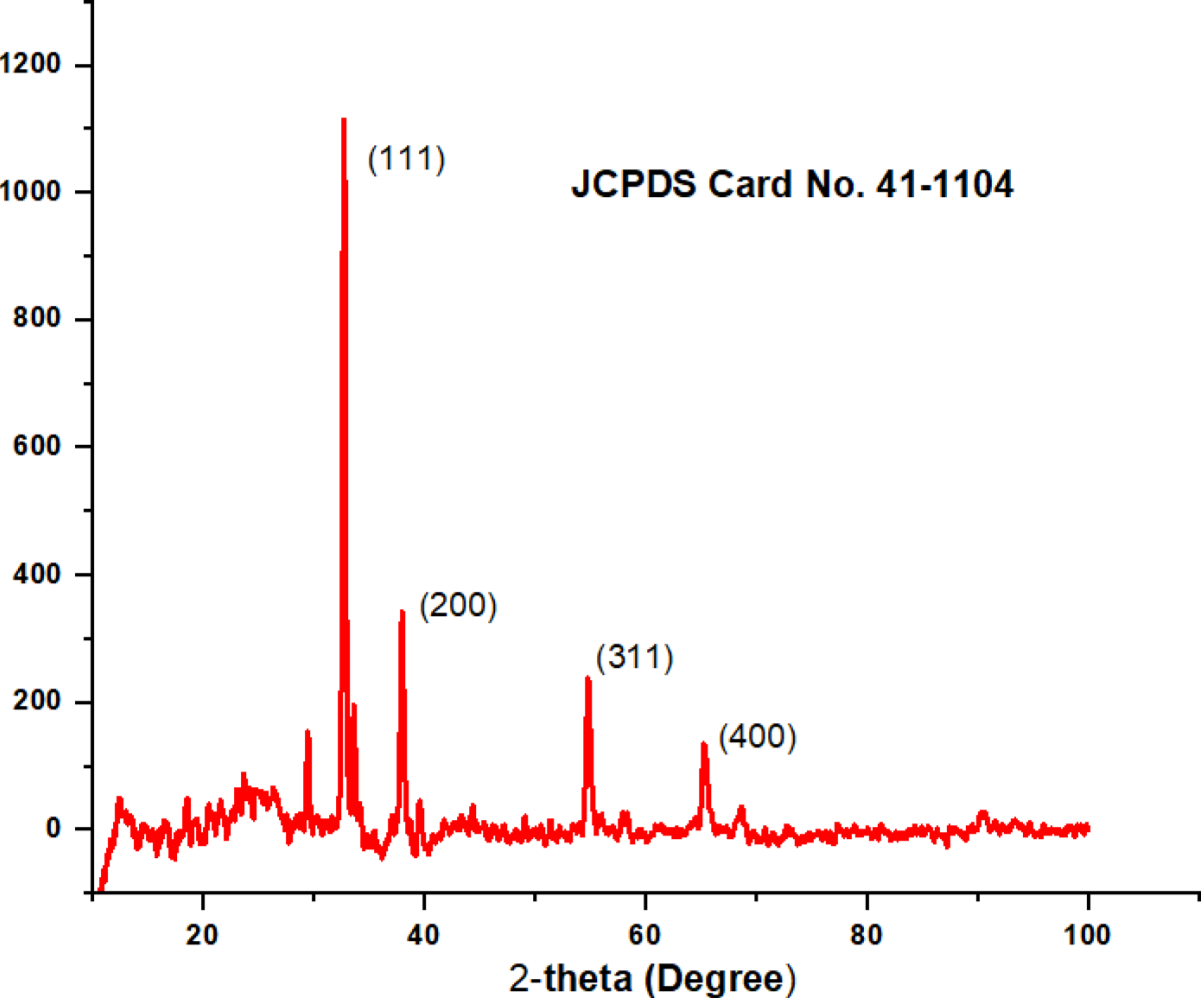
XRD structure of green synthesized Ag_2_ONPs.

The computed results revealed a microstrain value of 5.95$\:\times\:$10^−3^, a dislocation density of 2.33$\:\times\:$10^−3^ nm^− 2^, and a crystallinity degree of approximately 84%, indicating that the nanoparticles possess a high degree of structural order with minimal lattice imperfections (Table 2). See fig 2.

**Table 2:** XRD parameters of synthesized Ag_2_ONPs.

	Average Crystallite Size (D)	Microstrain (ε $\:\times\:$10^− 3^)	Dislocation Density (δ $\:\times\:$ 10^− 3^) (nm^− 2^)	Degree of Crystallinity
Ag_2_ONPs	20.7 nm	5.96	2.33	~ 84

### Morphological and elemental analysis (EM-EDX)

**Fig. 3:**
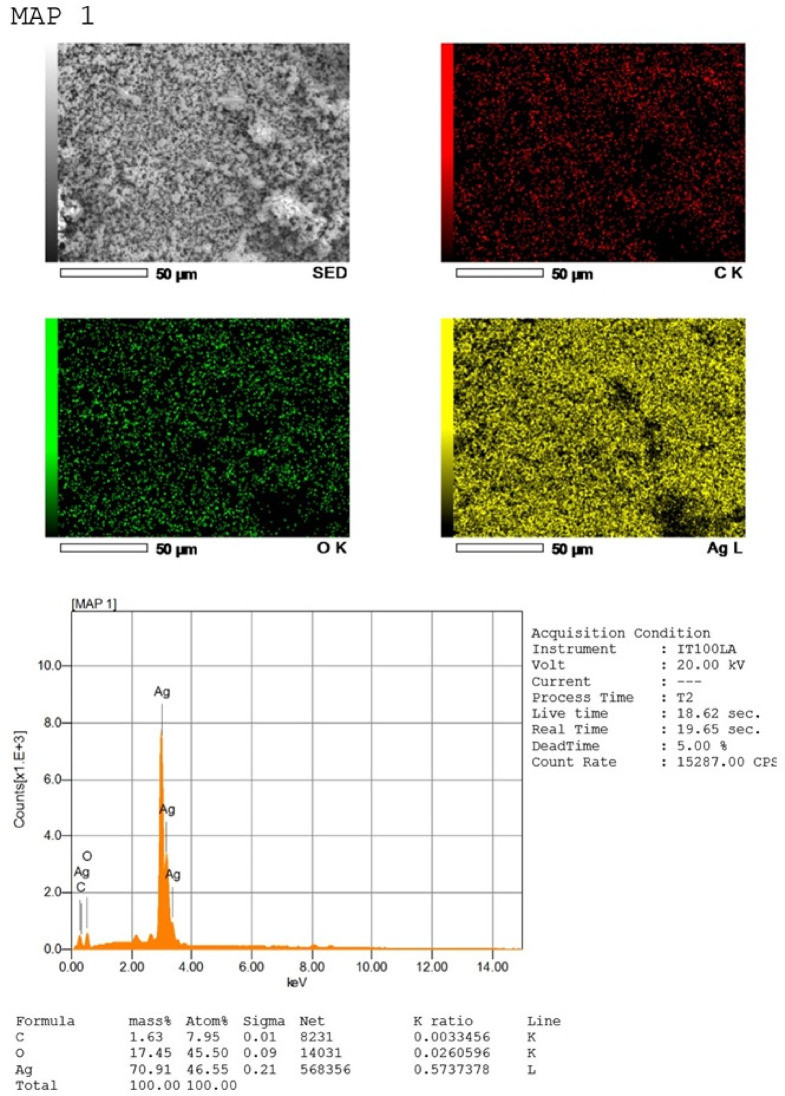
Elemental mapping and EDX- graph of green synthesized Ag_2_ONPs.

Elemental morphology and composition of the Ag_2_ONPs synthesized were studied through elemental mapping using Energy-Dispersive X-ray Spectroscopy (EDX) (Fig. 3). Elemental mapping was used to determine the distribution of elements present on the surface of the sample (Map 1). The maps indicate an even and continuous distribution of Silver (Ag) validating its successful and disseminated formation across the sample. Carbon (C) and oxygen (O) were also evenly distributed in the matrix. This is expected, as these substances are derived from organic biomolecules (including phenolics, flavonoids, and proteins), which serve as capping and stabilizing factors on the nanoparticle surface^57^. The EDX spectrum (Fig. 3) was used to qualitatively verify the elemental composition. The spectrum exhibits a strong and sharp optical absorption peak of silver (Ag) at approximately 3 keV, which is a typical signal of metallic silver nanoparticles. Elemental analysis indicated high purity of the sample, with 70.91% of the mass being silver. The remaining elements were oxygen (17.45%) and carbon (1.63%), confirming the presence of an organic capping layer from the plant extract, as has also been observed in other studies on green-synthesized nanoparticles^57^, ^58^.

### Scanning electron microscopy (SEM) analysis

**Fig. 4:**
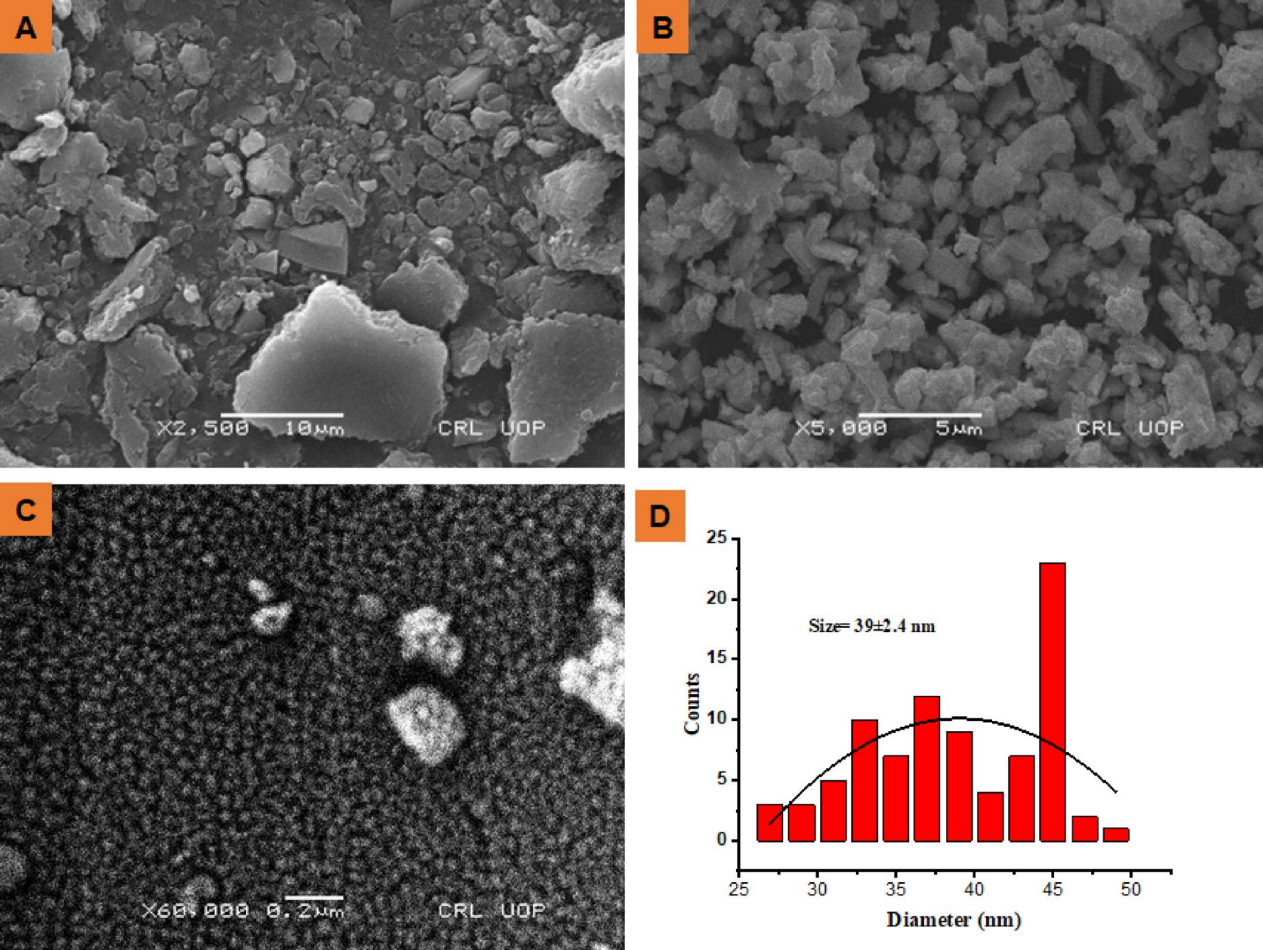
Scanning electron microscopy (SEM) analysis of the green-synthesized Ag_2_ONPs. At various magnifications: (**A**) 2,500x magnification - showing agglomerates. (**B**) 5,000x magnification. (**C**) High-magnification (60,000x) image displaying the quasi-spherical morphology of individual nanoparticles. (**D**) Particle size distribution histogram (39 ± 2.4 nm).

Scanning Electron Microscopy (SEM) was used to study the surface morphology and size of the synthesized Ag_2_ONPs and the results are shown in Fig. 4. In Fig. 4C, the high-magnification SEM micrograph of the synthesized nanoparticles clearly shows that the particles are well-defined and have a quasi-spherical shape. Although the particles cluster into larger clusters at low magnifications (Fig. 4A and B), the high-resolution image reveals that these clusters consist of individual primary nanoparticles that do not merge. This observation shows that plant extract phytochemicals act as effective capping agents, inhibiting aggregation at the primary-particle level. Particle size distribution analysis was conducted based on the SEM images to determine the average size of the nanoparticles. The histogram in Fig. 4D shows that the particle diameters range from approximately 25 to 50 nm. From this analysis, the average size of the Ag_2_ONPs was calculated to be 39 ± 2.4 nm. These results confirm the successful synthesis of nanoparticles with diameters below 100 nm. The SEM analysis verifies the formation of well-defined, quasi-spherical Ag_2_ONPs with a consistent size distribution.

### Zeta potential analysis

Zeta potential (ZP) analysis was used to determine the stability and surface charge of the synthesized Ag_2_ONPs. The ZP is a key parameter for assessing the stability of colloidal dispersions, as it reflects the level of electrostatic repulsion between similarly charged particles in solution. Generally particles, with ZP values exceeding + 30 mV or below − 30 mV, are considered highly stable, because the repulsive forces are powerful enough to prevent aggregation^59^. Figure 4A shows the zeta potential distribution of the biosynthesized Ag_2_ONPs. A sharp peak was observed, with an average ZP of -39 mV and a standard deviation of 1.8 mV. This highly negative value is well beyond the typical stability threshold of -30 mV, indicating that the Ag_2_ONPs are highly charged on their surfaces. This repulsive force generates strong electrostatic repulsion between particles, preventing aggregation and enabling the formation of a stable colloidal dispersion. Recently, AgNPs synthesized from *Withania coagulans* were reported to have an average particle diameter of 26.63 nm, crystalline nature, and stability (zeta potential of -21.4 mV),^43^ which closely aligns with our findings. This supports that Ag_2_ONPs were negatively charged and evenly distributed in the solution.

### Dynamic light scattering (DLS) for size distribution

**Fig. 5:**
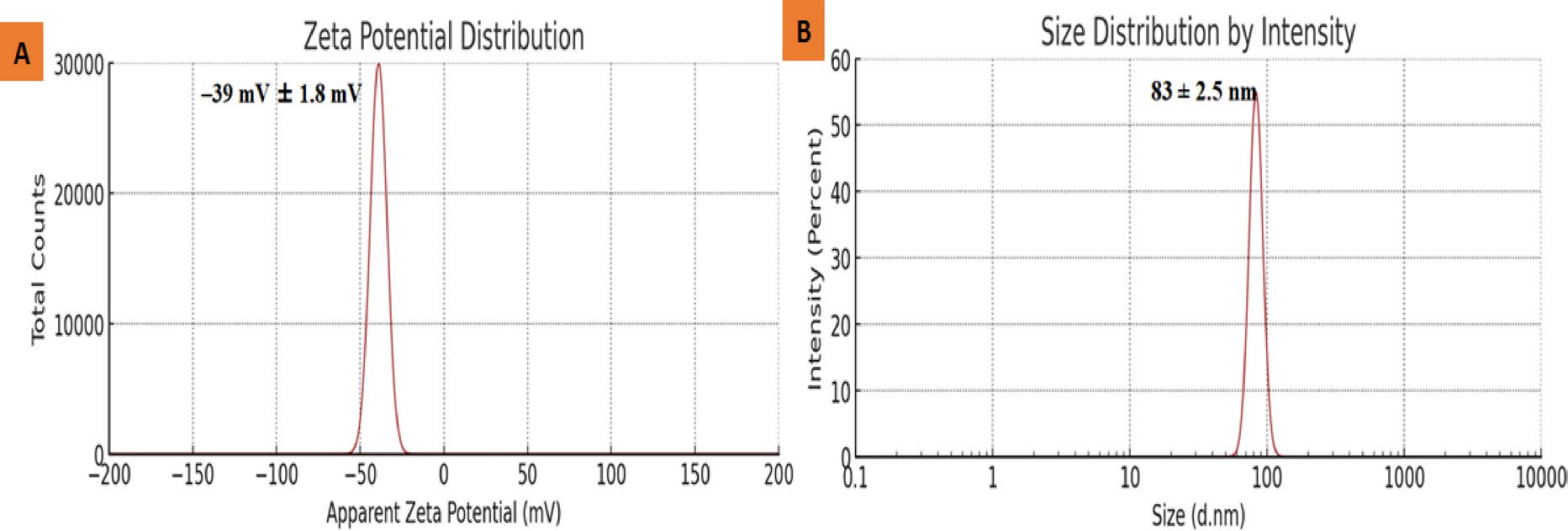
Characterization of synthesized Ag_2_ONPs. (**A**) Zeta potential distribution curve, showing a mean surface charge of -39 mV ± 1.8 mV, which indicates high colloidal stability. (**B**) Hydrodynamic size distribution measured by Dynamic Light Scattering (DLS), revealing an average particle diameter of 83 nm ± 2.5 nm.

Dynamic light scattering (DLS) was used to measure the average hydrodynamic size and size distribution of the Ag_2_ONPs. This method measures the size of particles in a colloidal suspension, including the core particles and any solvent or surface layers. Figure 4B shows the size distribution profile, showing particle intensity versus particle diameter. The average hydrodynamic diameter of Ag_2_ONPs was found to be 83 nm ± 2.5 nm. The presence of a single narrow peak in the DLS graph indicates that the nanoparticle population is monodisperse and uniform in size. This hydrodynamic size represents the effective size of the NPs in solution, and is essential for understanding their possible interactions in biological or environmental systems^30^ see fig 5.

### Thermogravimetric and differential scanning calorimetry (TGA/DSC) analysis

**Fig. 6:**
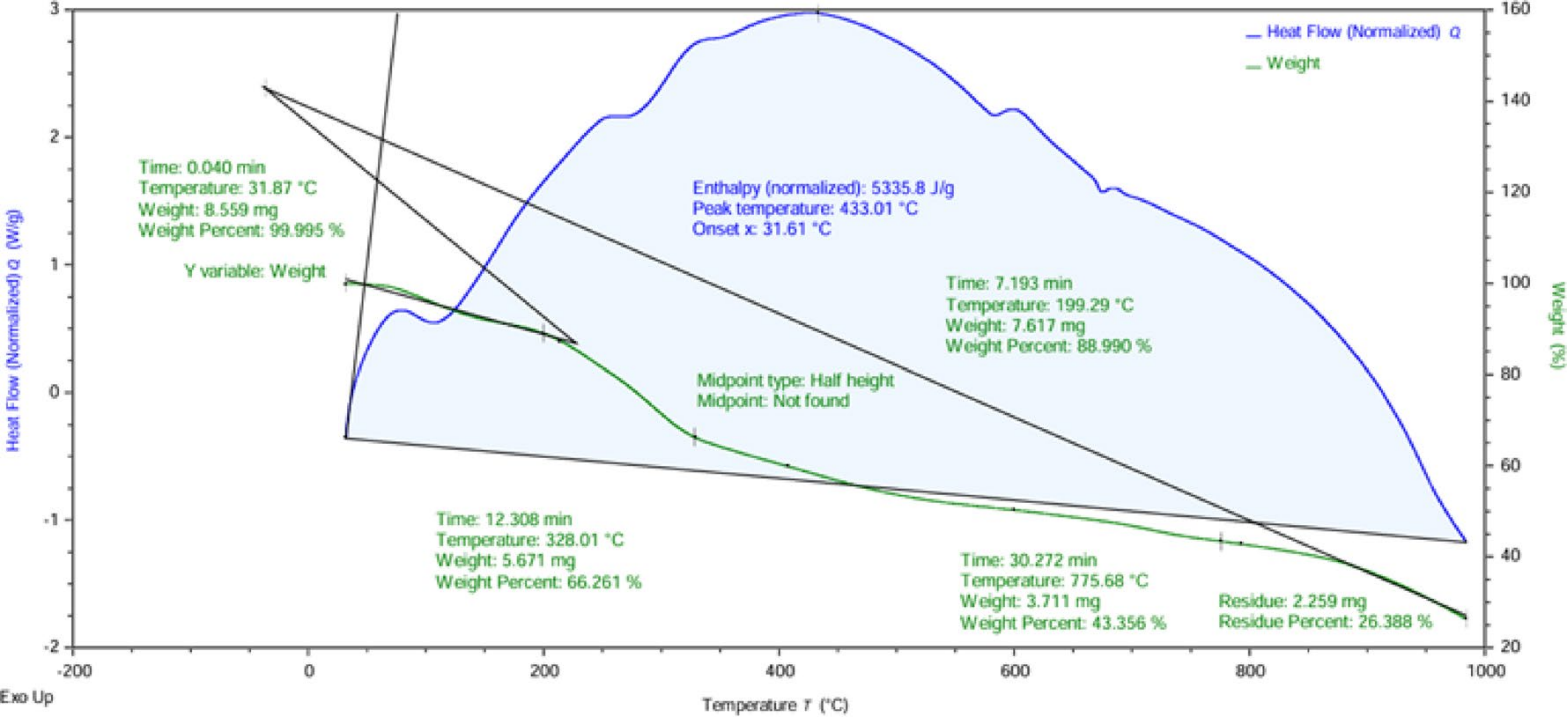
TGA/DSC thermogram of the green-synthesized Ag_2_ONPs. The TGA curve (green) shows the percentage weight loss, and the DSC curve (blue) shows the corresponding heat flow as a function of temperature. The major exothermic peak at 433.01 °C corresponds to the decomposition of organic capping agents.

The thermal stability, compositional characteristics, and decomposition behavior of the green-synthesized Ag_2_ONPs were examined using thermogravimetric analysis (TGA) combined with differential scanning calorimetry (DSC). The thermal profile revealed a typical multi-step degradation pattern, indicating the presence of phytochemical residues from the *N. cataria* extract acting as stabilizing and capping agents (Fig. 6). The TGA curve displayed mass loss in three consecutive stages. The initial phase, occurring between 30 °C and approximately 200 °C, exhibited a slight reduction of approximately 11%, attributed to the removal of adsorbed water and volatile components loosely bound to the nanoparticle surface. This trend is typical for plant-based nanomaterials with physically bonded water molecules.

The most intense and quick reduction in mass was noted at temperatures between 200 °C and 600 °C which is the main stage of decomposition. This significant loss is associated with the disintegration and oxidative diminution of organic biomolecules derived from *N. cataria* which serve as reducing, capping, and stabilizing agents. These organic elements are burned; therefore, most of the overall weight loss is attributed to their combustion. The reduction in mass was slower and more gradual beyond 600 °C, presumably because of the removal of remaining carbonaceous material and the removal of more firmly bound organic fragments still present on the surface of the nanoparticle.

The TGA results were supported by a DSC thermogram. The wide endothermic signal observed at temperatures below 100 °C validated the energy acquired during moisture evaporation. This was followed by a strong exothermic peak at 433.01 °C associated with the combustion of organic constituents on the surface. The exothermic transition showed an enthalpy change of 5335.8 J/g indicating that a large amount of heat was produced during organic decomposition. By the end of the heating process (~ 950 °C), 26.39% of the original mass remained as a stable residue. This remaining fraction is the inorganic silver oxide core, demonstrating its thermal stability after the removal of all organic substances. Although silver oxide usually decomposes at high temperatures, this change can be masked by overlapping organic incineration events in the compound’s thermal profile.

Overall, the TGA/DSC findings confirm that Ag_2_ONPs were successfully fabricated as a thermally stable inorganic core with a significant organic phytochemical shell. The active nature of the green synthesis process and the strong thermal stability of the resulting nanomaterial were demonstrated by the slow decay of organic matter and the maintenance of a stable residual mass.

### Biological activities

#### Antibacterial assay

**Fig. 7:**
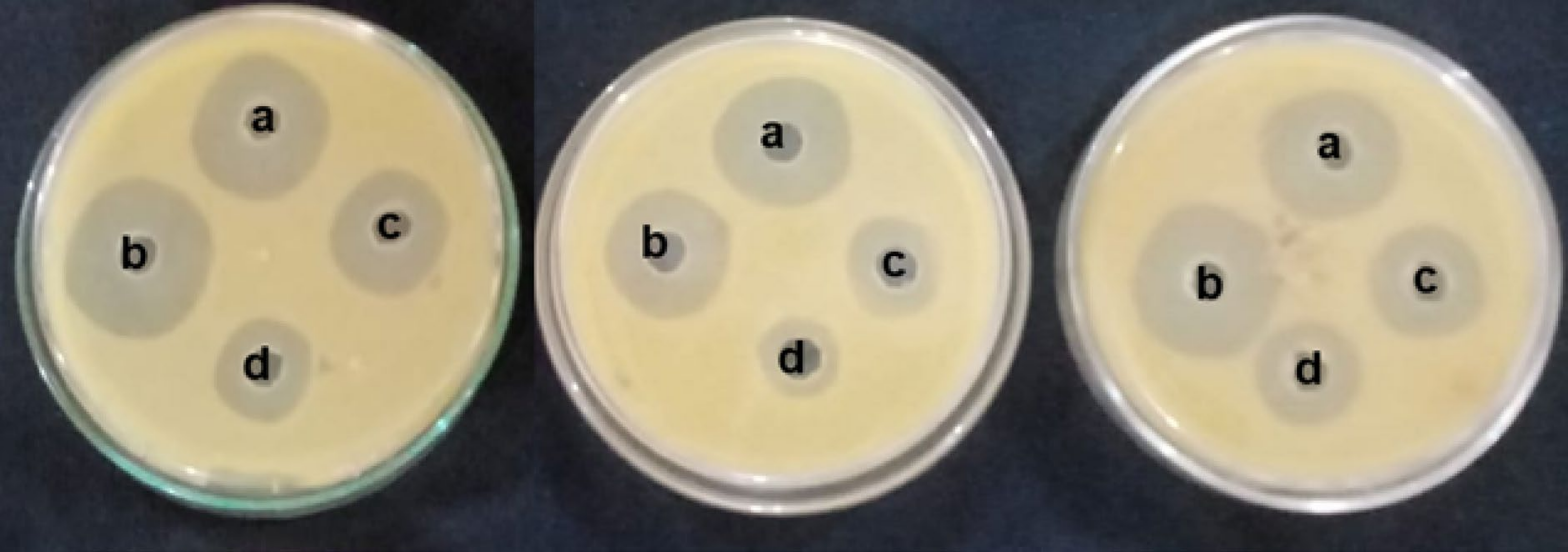
Antibacterial Potential of Ag_2_ONPs against; (A)* S. aureus* (B)* B. subtilis* (C)* E. coli* respectively. In each plate, the wells correspond to: (**a**) Biosynthesized Ag₂ONPs (30 µg/mL), (**b**) Positive Control (Gentamicin, 30 µg/mL), (**c**) Biosynthesized Ag₂ONPs (15 µg/mL), and (**d**) *N. cataria* L. flowers extract (30 µg/mL).

**Fig. 8:**
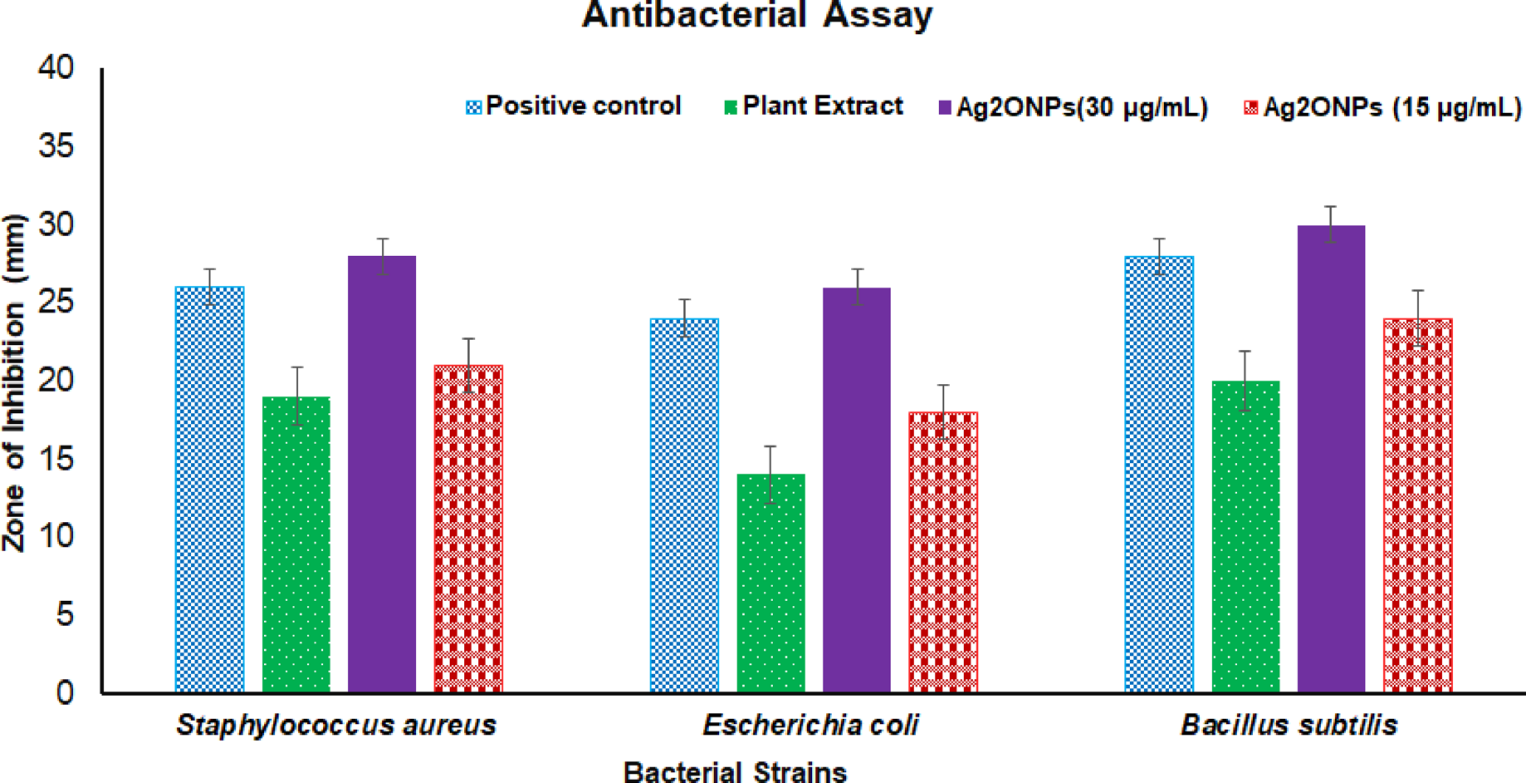
Antibacterial activity of Ag_2_ONPs against test bacterial strains by agar well diffusion method. Values represent Mean ± SD (*n* = 3). It is necessary to use different letters to show that the differences are significant (Tukey HSD, (*P* ≤ 0.05)).

The antibacterial activity of the synthesized Ag_2_ONPs was tested against Gram-positive (*S. aureus*,* B. subtilis*) and Gram-negative (*E. coli*) bacteria using the agar well diffusion technique. The findings, shown as the zone of inhibition in millimeters (mm), are presented in Fig. 7. The quantitative effects, measured as the Zone of Inhibition (ZOI) are presented in Table 3 and represented graphically in Fig. 8. The Ag_2_ONPs exhibited strong dose-dependent antimicrobial activity. Statistical analysis (one-way ANOVA followed by Tukey HSD test) showed that a high concentration of Ag_2_ONPs (30 µg/mL) produced significantly bigger inhibition zones than the lower concentration (15 µg/mL) and the crude plant extract (*P* ≤ 0.05).

The 30 µg/mL Ag₂ONPs treatment showed superior bactericidal activity compared with to positive control (Gentamicin) against all tested strains. The greatest inhibition was observed against *B. subtilis* (30.00 ± 0.51 mm), *S. aureus* (28.00 ± 0.43 mm), and *E. coli* (26.00 ± 0.53 mm). Statistical grouping, represented by different superscript lowercase letters (a–d) in Table 3 confirms that the efficacy of the synthesized nanoparticles at 30 µg/mL was significantly higher than that of the antibiotic control.

The MIC values further confirmed the potent bactericidal effect of Ag₂ONPs, with 7.5 ± 0.5 µg/mL against *B. subtilis*, 10.0 ± 0.2 µg/mL against *S. aureus*, and 12.5 ± 0.8 µg/mL against *E. coli*, which were lower than those of the crude plant extract and comparable to Gentamicin (Table 4). These findings strongly align with recent literature on green-synthesized silver oxide nanoparticles. For instance, Zúñiga-Miranda et al. (2024) investigated the bioactivity of silver oxide nanoparticles and reported significant antibacterial efficacy against both *S. aureus* (ATCC 25923) and *E. coli* (ATCC 25922). A minimum inhibitory concentration (MIC) of 22.5 ug/mL was determined for both strains in their study^60^. Likewise, silver oxide nanoparticles produced using *Borassus flabellifer* L. Fiber extract demonstrated strong antibacterial activity against dental pathogens such as *E. coli* and *B. subtilis* etc. ^61^.

**Table 3:** Antibacterial activity of green-synthesized Ag_2_ONPs compared to controls.

Treatments	*S. aureus* (mm)	*B. subtilis* (mm)	*E. coli* (mm)
Ag₂ONPs (30 µg/mL)	28.00 ± 0.43^a^	30.00 ± 0.51^a^	26.00 ± 0.53^a^
Positive Control (Gentamicin, 30 µg/mL)	26.00 ± 0.43^b^	28.00 ± 0.51^b^	24.00 ± 0.53^b^
Ag₂ONPs (15 µg/mL)	21.00 ± 0.43^c^	24.00 ± 0.51^c^	18.00 ± 0.53^c^
Plant Extract	19.00 ± 0.43^d^	20.00 ± 0.51^d^	14.00 ± 0.53^d^

Values are given as mean ± SD(*n* = 3) Letters in different superscripts (^a^,^b^,^c^,^d^) within the same column show statistically significant differences between treatments based on the Tukey HSD test with *P* ≤ 0.05.

**Table 4:** Minimum inhibitory concentration (MIC) and Minimum bactericidal concentration (MBC) of AgO_2_NPs, plant extract and positive control against Gram-positive and Gram-negative bacterial strains.

Treatment	*B. subtilis* MIC (µg/mL)	*S. aureus* MIC (µg/mL)	*E. coli* MIC (µg/mL)
Plant Extract	150.0 ± 2.0^a^	180.0 ± 2.0^a^	250.0 ± 4.0^a^
Ag₂ONPs	7.5 ± 0.5^b^	10.0 ± 0.2^b^	12.5 ± 0.8^b^
Positive Control (Gentamicin)	5.0 ± 0.2^b^	8.0 ± 0.5^b^	10.0 ± 0.4^b^

Values represent Mean ± SD (*n* = 3). Different superscript letters (^a^, ^b^) indicate statistically significant differences (*P* < 0.05) based on Tukey’s HSD test.

**Fig. 9:**
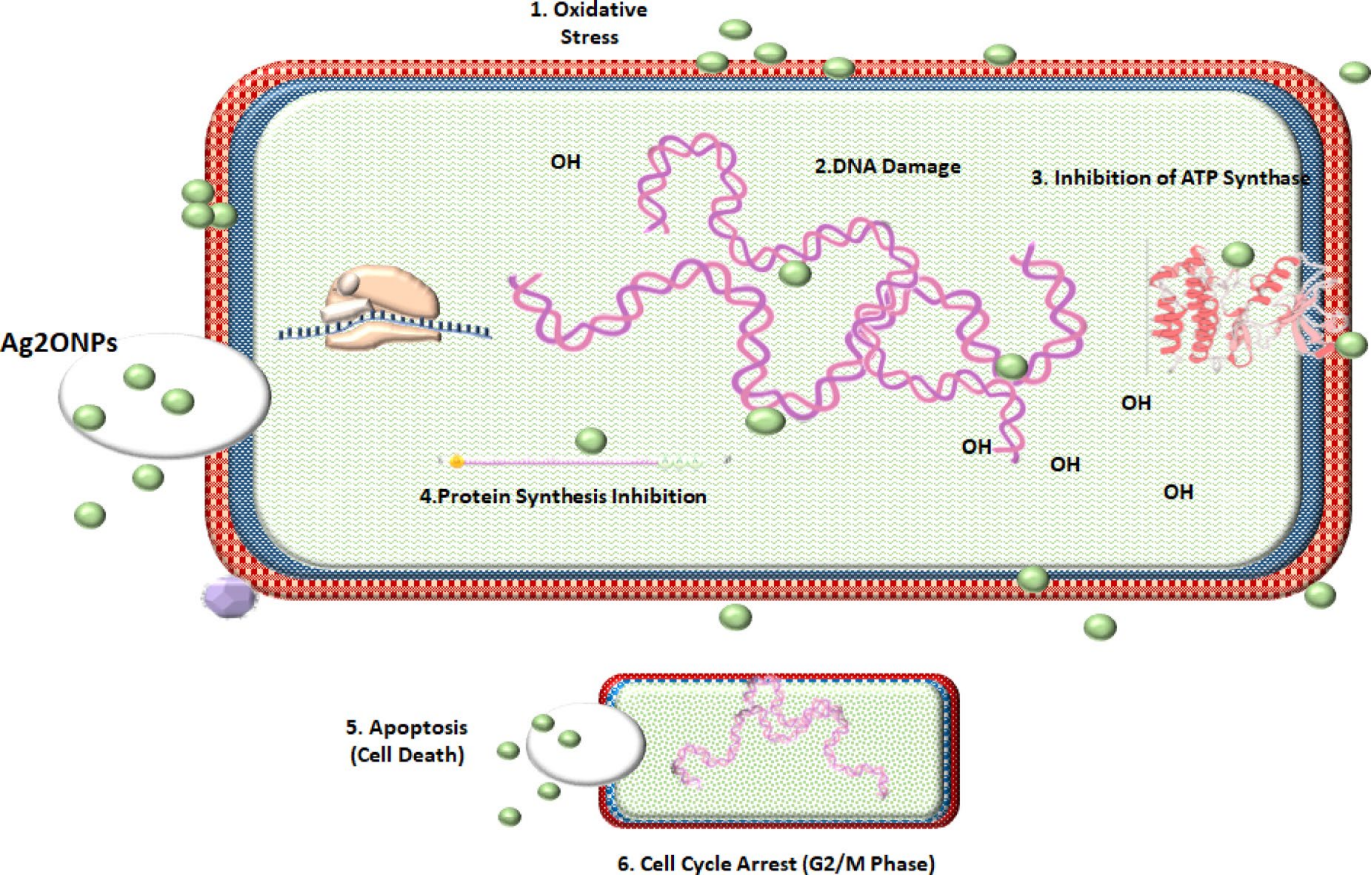
Supposed antibacterial mechanism of Ag_2_ONPs.

The potent, broad-spectrum activity of the synthesized Ag_2_ONPs against both gram-positive and gram-negative bacteria can be attributed to the well-established mechanisms of silver nanoparticles^62^. These mechanisms include the release of silver ions (Ag), which disrupt cell membrane integrity, inhibit respiratory enzymes, and interact with sulfur-containing proteins and DNA, ultimately leading to bacterial cell death. The superior performance of the nanoparticles compared to the plant extract alone suggests a potent synergistic effect. This enhanced activity is likely due to the combined action of the Ag_2_ONPs core and the bioactive phytochemicals capping the surface^62,63^. In contrast to many previous studies on silver nanoparticles (AgNPs), the Ag_2_ONPs synthesized in this work using Nepeta cataria flower extract demonstrated clear advantages for antibacterial applications. Green synthesis methods using plant extracts are widely documented as environmentally friendly, cost‑effective, and less toxic compared to conventional chemical methods, which often involve hazardous reducing agents and stringent conditions (e.g., plant‑mediated green synthesis: reactive phytochemicals reduce toxicity and environmental burden)^64,65^. Moreover, many studies on AgNPs show significant antibacterial activity against various pathogens, but often focus solely on in vitro inhibition zones without deeper mechanistic insights^66^. In our study, the Ag₂ONPs exhibited potent antibacterial activity with promising inhibition zones against both gram‑negative and gram‑positive bacteria, outperforming the plant extract alone and aligning with reports that green‑synthesized silver nanomaterials show stronger antimicrobial effects than extracts or chemically synthesized analogs^67^. Importantly, we also incorporated in silico molecular docking analysis to provide additional evidence for specific nanoparticle–protein interactions, offering mechanistic insights often lacking in previous reports focused only on empirical antibacterial assays. Together, these features position our Ag_2_ONPs as a highly effective, sustainable, and biologically insightful platform for antibacterial applications. Our findings are also in close agreement with those reported in the literature^25^. Figure 9 illustrates the antibacterial mechanism of Ag_2_ONPs.

#### Antifungal assay

**Fig. 10:**
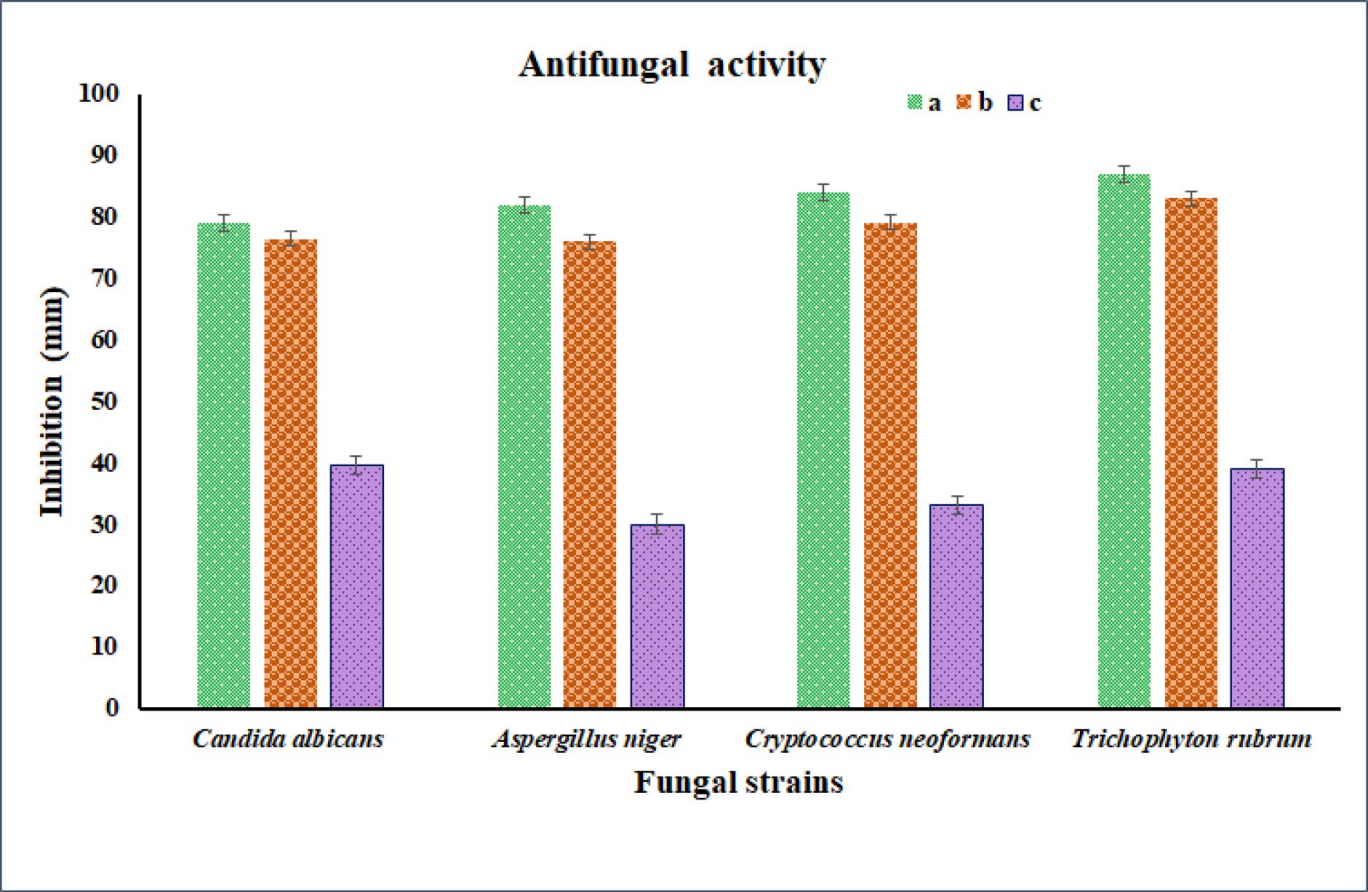
Antifungal activity of green synthesis of Ag_2_ONPs: The bar chart indicates the mean zone of inhibition (mm) of (**a**) positive control (terbinafine, 30 µg/mL), (**b**) green-synthesized Ag_2_ONPs (30 µg/mL) and (**c**) *N. cataria* L. flowers extract (30 µg/mL) on four clinically relevant fungal strains; *C. albicans*,* A. niger*,* C. neoformans* and *T. rubrum* was utilized. The one-way ANOVA test showed that the inhibition profile of the treatments was significantly different (*P* < 0.001). Follow-up post-hoc testing was done using Tukey HSD test which showed that Ag_2_ONPs were a significantly more effective agent compared to the crude extract (*P* ≤ 0.05) and statistically similar to the positive control in most strains.

Pathogenic fungi pose a significant and often underestimated threat to global health. Each year, fungal infections cause over 1.6 million deaths worldwide, a mortality rate that rivals tuberculosis, and is approximately three times higher than that of malaria^68^. This challenge is further compounded by the steady rise of antifungal-resistant strains, which severely limits treatment options. Consequently, there is an urgent need to develop novel therapeutic agents Fig. 10 indicates that the antifungal activity of three substances i.e.: a (positive control), b (Ag₂ONPs) and c (*N. cataria* extract) was quantitatively evaluated against a panel of four pathogenic fungi i.e.: a, b, and c. *C. albicans*,* A. niger*,* C. neoformans* and *T. rubrum* were selected as fungal strains. As shown in Fig. 10; Table 5, the inhibition process varied significantly among treatments (one-way ANOVA, *P* < 0.001). The positive control showed antifungal activity, with inhibition zones varying between 79 and 87 mm. Notably, the biosynthesized Ag₂ONPs demonstrated stronger antifungal activity than the standard drug, with inhibition zones measuring between 76 and 83 mm. The crude *N. cataria* extract also showed measurable antifungal activity (30–40 mm), supporting its potential as a plant-based antifungal agent.

The post-hoc analysis using the Tukey HSD test revealed that Ag_2_ONPs activity was significantly greater than that of the crude extract (*P* ≤ 0.05) in all tested strains. *T. rubrum* and *C. neoformans* were the most susceptible to Ag_2_ONPs treatment. The experimental precision and reproducibility were high, as demonstrated by the low coefficient of variance (CV < 2%) and uniform mean ± SD values (*n* = 3). These results indicate that Ag_2_ONPs exhibit broad antifungal activity, making them a promising alternative for antifungal therapeutic development. The investigation confirms that Ag_2_ONPs possess potent antifungal properties that are decidedly superior to those of the crude extract and comparable to the standard positive control. This potent bioactivity suggests that the active components of plant extracts can also effectively disrupt the viability of diverse fungal pathogens^69^, highlighting their potential for further development as antifungal agents.

**Table 5:** Antifungal activity of different treatments against fungal strains (zone of inhibition, mm; Mean ± SD, *n* = 3).

Treatments	*C. neoformans*	*C. albicans*	*A. niger*	*T. rubrum*
Positive Control	79.00 ± 1.00^a^	82.00 ± 1.00^a^	84.00 ± 1.00^a^	87.00 ± 1.00^a^
Ag₂ONPs	76.00 ± 1.00^b^	76.00 ± 1.00^b^	79.00 ± 1.00^b^	83.00 ± 1.00^b^
*N. cataria* L. extract	40.00 ± 1.00^c^	30.00 ± 1.00^c^	33.00 ± 1.00^c^	39.00 ± 1.00^c^

Values are expressed as the mean ± SD. Different superscript letters (^a^,^b^,^c^) within a column indicate significant differences according to Tukey’s HSD test at *P* ≤ 0.05.

#### In vitro hemolytic potential of green synthesized Ag_2_ONPs

The hemolytic activity of the biosynthesized Ag_2_ONPs was systematically determined by observing the optical density (OD) at 540 nm using different concentrations. The control conditions were phosphate-buffered saline (PBS) (OD = 0.11), and Triton X-100 (TX-100) (OD = 2.50), representing 0% and 100% hemolysis, respectively.

At concentrations of 0.5 to 3.5 µg/mL, Ag_2_ONPs showed low hemolytic activity, with percentage hemolysis ranging from 0.19 to 3.88% (Table 6). The findings indicated that hemolysis was concentration-dependent as the nanoparticle dose increased. These results are consistent with a recent study by Al-Asbahi et al. ^53^, who concluded that biogenic silver-silver oxide nanocomposites are very biocompatible, exhibiting a low level of hemolysis (1.8%) at a lower concentration.

As a hemolysis rate of less than 5% is usually acceptable for biomaterials, Ag_2_ONPs may be considered hemocompatible within the tested concentration range (3.5 µg/mL and below). Their low hemolytic activity at lower concentrations indicates high biocompatibility and implies that these nanoparticles are relatively safe for biomedical use. Nevertheless, the increased hemolysis observed at higher concentrations could pose a risk of erythrocyte membrane damage and should be taken seriously in clinical or systemic applications.

Thus, the dose-dependent hemolytic profile highlights the significance of optimizing the concentration to ensure that Ag_2_ONPs are safe and effective for biomedical treatments and diagnosis, especially when close contact with blood is expected. These results are consistent with other reports on the excellent hemocompatibility of Ag_2_ONPs^70^.

**Table 6:** Hemolytic activity of biosynthesized Ag₂ONPs (OD at 540 nm and % Hemolysis).

Sample Concentration (µg/mL)	OD at 540 nm	% Hemolysis
0.5	0.119	0.19
1.0	0.121	0.29
1.5	0.124	0.39
2.0	0.163	2.03
2.5	0.172	2.45
3.0	0.181	2.88
3.5	0.19	3.88

#### Anticoagulant and thrombolytic activity of Ag_2_ONPs

**Fig. 11:**
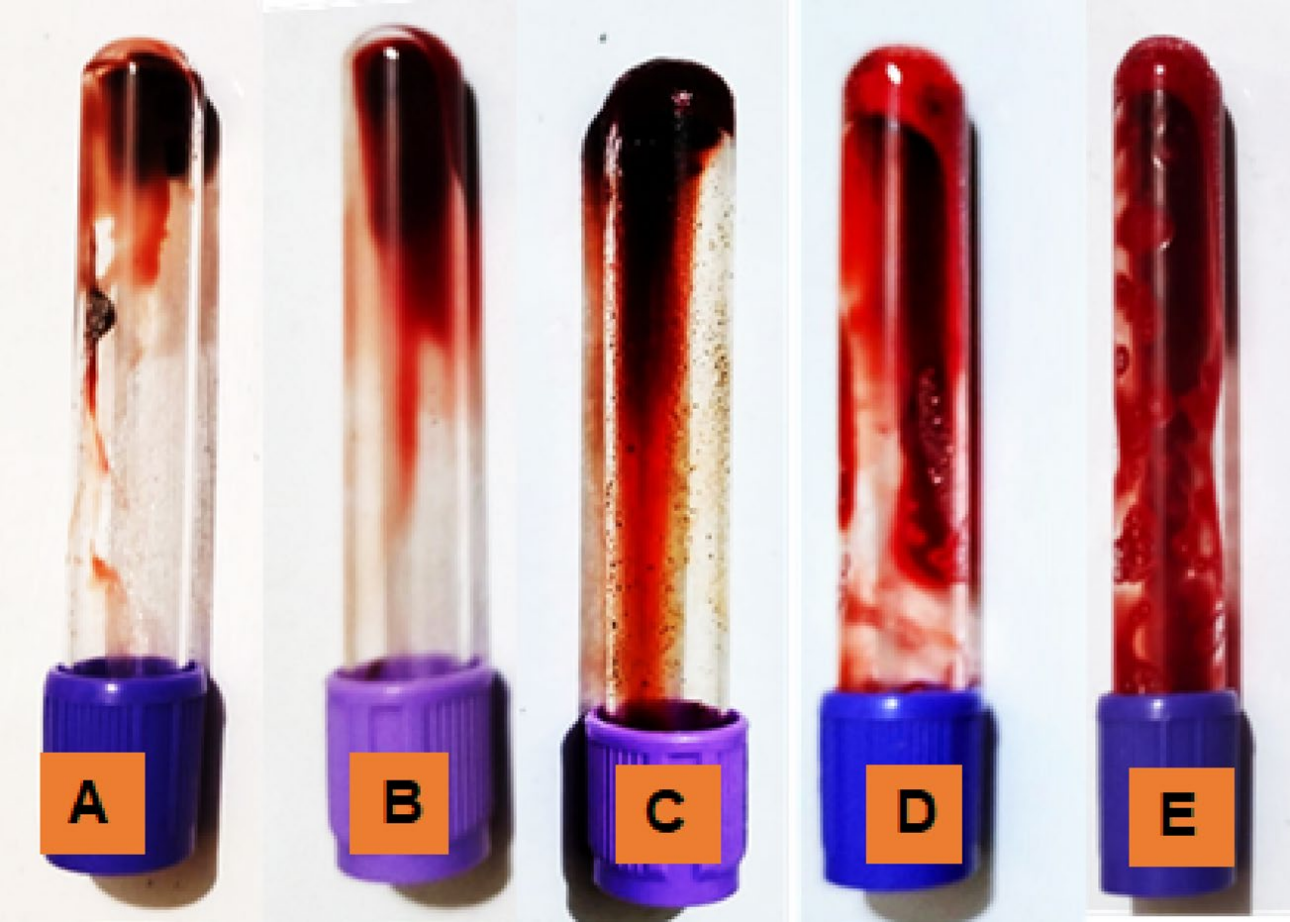
Dose-dependent anticoagulant activity of Ag_2_ONPs. The labels correspond to the following concentrations of Ag_2_ONPs: (**A**) Negative control (**B**) 10 µg/mL, (**C**) 20 µg/mL, (**D**) 30 µg/mL, and (**E**) 40 µg/mL.

The anticoagulant activity of the green-synthesized Ag_2_ONPs was intended by testing human blood incubated with different concentrations of the nanoparticles (10–40 µg/mL). A dose-dependent delay in coagulation was observed, as shown in Fig. 11. A clear visual trend demonstrated that, as the concentration of Ag_2_ONPS increased, blood clotting progressively decreased. In Tube A (negative control) and Tube B (10 µg/mL), the blood samples were fully coagulated and formed a solid clot. But as the concentration of nanoparticles increased in Tubes C and D, clot formation was increasingly inhibited. Tube E (40 µg/mL), showed full inhibition of the coagulation cascade, and all the blood was kept in liquid form. These results demonstrate that the anticoagulant effect of Ag_2_ONPs is dose-dependent, meaning that higher concentrations of nanoparticles are more effective in preventing fibrin mesh formation and subsequent blood clotting.

**Fig. 12:**
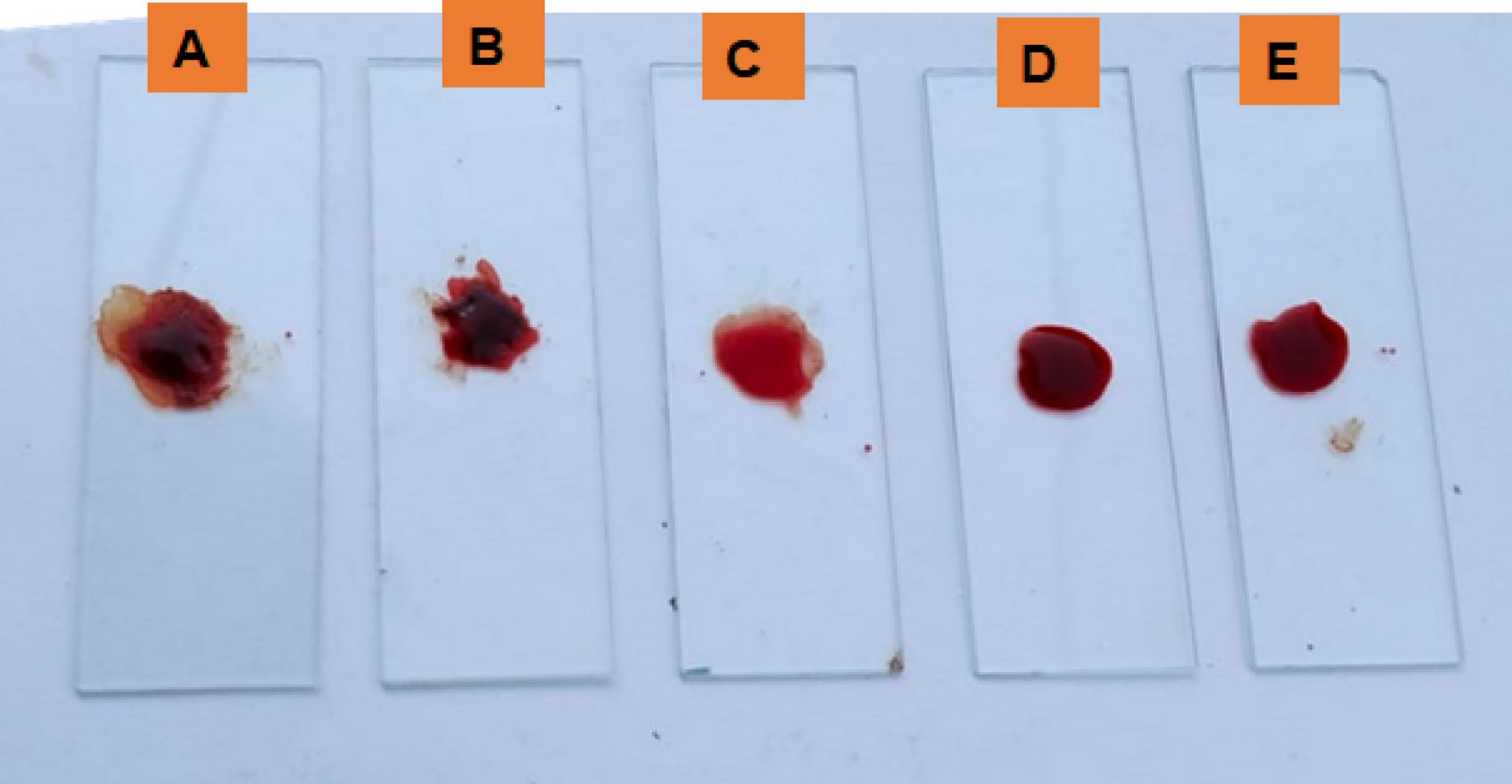
Dose-dependent thrombolytic activity of Ag_2_ONPs at various concenrtaions (**A**) Negative Control (without Ag_2_ONPs), (**B**) 10 µg/mL, (**C**) 20 µg/mL, (**D**) 30 µg/mL, and (**E**) 40 µg/mL.

The thrombolytic potential of Ag_2_ONPs was further investigated using pre-formed blood clots placed on glass slides, as shown in Fig. 12. Visible dose-dependent clot degradation was recorded, with Sample E (highest concentration) showing the most extensive clot lysis, followed by a gradual increase in clot dissolution from Samples B to D (lower concentrations). While Sample A (negative control) remained a solid untreated clot. These findings indicate that Ag_2_ONPs have a powerful thrombolytic effect, which may be mediated through interference with the fibrin meshwork or stimulation of native plasminogen pathways. These concentration-dependent anticoagulant and thrombolytic properties are consistent with previously documented literature on metallic nanoparticles, which reveal interference with the prothrombin-to-thrombin transformation cascade and increased fibrinolytic activity^71^. The slide- and tube-based assays used in this study served as preliminary qualitative screening methods to observe visible clot inhibition and clot lysis behavior of Ag_2_ONPs, rather than as standardized clinical coagulation assessments such as PT/INR. Anticoagulants primarily inhibit clot formation and propagation rather than directly dissolving pre-formed clots. Therefore, the observed clot reduction may be associated with nanoparticle-mediated interference with fibrin structure or facilitation of endogenous fibrinolytic processes. Comprehensive quantitative coagulation analyses, including PT/INR and other plasma-based assays, were not performed due to limited access to specialized clinical laboratory facilities during the study period and will be considered in future investigations for clinical validation. In general, these findings imply that green-synthesized Ag_2_ONPs are not only biocompatible but also have therapeutic potential as anticoagulants or thrombolytic nanomedicines in blood-contact applications.

#### In silico molecular docking analysis

**Fig. 13:**
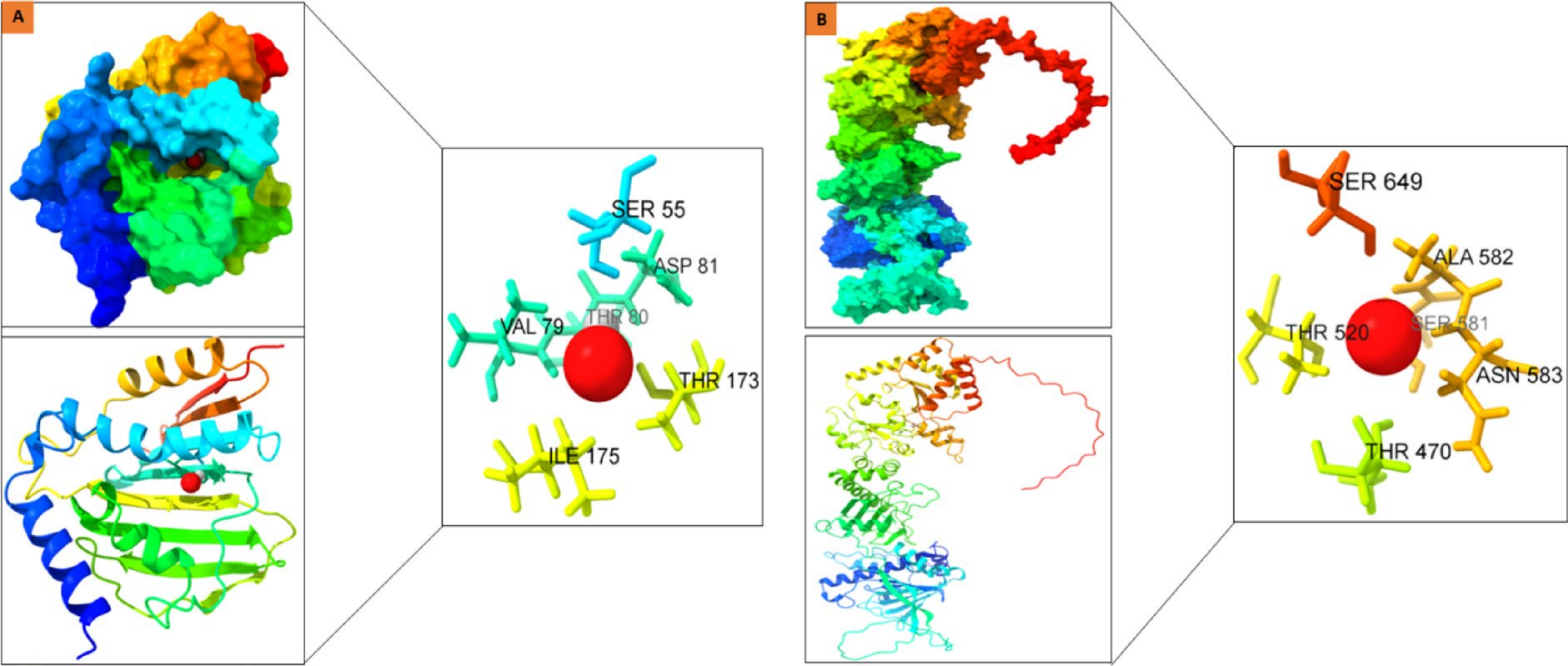
(**A**) Bacterial protein, DNA gyrase subunit B from *S. aureus*, including its PDB database identifier (3U2D) (**B**) fungal protein, Hsp90-like protein from *T. rubrum*, including its AlphaFold database identifier (AF-F2SVB4-F1-v6).

To further investigate the potential mechanism underlying the observed potent antimicrobial activity, in silico molecular docking was performed. The synthesized Ag₂ONPs were docked against key proteins from selected pathogenic bacteria and fungi. The docking scores, representing the binding affinity between the nanoparticles and proteins, are presented in Table 7. Notably, all interactions resulted in favorable negative docking scores, suggesting that the nanoparticles can form stable and spontaneous complexes with these crucial microbial proteins (Table 1S and Fig. 1S). To illustrate these findings, we highlight the interactions between one key bacterial target and one fungal target. Among the bacterial proteins, the strongest binding affinity was predicted for DNA gyrase subunit B from *Staphylococcus aureus*, which yielded a docking score of -2.50169 kcal/moL. This enzyme is essential for DNA replication and maintenance in bacteria. A strong interaction within its active site, as predicted by this docking score and visualized in Fig. 13A, suggest that the AgO-NPs could effectively inhibit this critical process. DNA gyrase inhibition is an effective bactericidal mechanism; DNA replication, and triggers the production of cytotoxic reactive oxygen species (ROS), resulting in oxidative cell death^72^. This mechanism aligns with findings that AgNPs induce lethal DNA damage via oxidative stress^73^, providing a clear molecular basis for the potent bactericidal activity observed against *S. aureus*. This is consistent with recent extensive reviews showing that AgNPs exert strong bactericidal effects on multidrug-resistant Gram-positive and Gram-negative bacteria associated with the disruption of crucial cellular membranes and enzymatic activities^74^. The key amino acid interactions predicted in our docking model (Fig. 13A) are strongly supported by previous in silico studies. Our finding that Aspartic Acid (ASP 81) is a primary contact point for AgO-NPs directly agrees with studies identifying Aspartic Acid as a crucial residue for anchoring silver nanoparticles to bacterial proteins^75,76^.

Similarly, within the fungal panel, AgO-NPs demonstrated a particularly strong interaction with the Hsp90-like protein from *T. rubrum*, with a docking score of -2.45627 kcal/mol. Hsp90 is a vital molecular chaperone required for the fungal stress response, morphogenesis, and viability, making it an attractive antifungal target. This strong predicted binding affinity, shown in Fig. 13B, aligns with our in vitro results, where the nanoparticles exhibited the highest antifungal activity against *T. rubrum*. This mechanism is supported by recent literature recognizing Hsp90 as an important therapeutic target; blocking this chaperone interferes with the folding of vital proteins needed by fungi to survive and cause disease^77^. This suggests that the potent anti-trichophyton effect may result from disruption of Hsp90 function. While the binding interactions for other targets are detailed in Table 6, these two examples demonstrate that green-synthesized Ag₂ONPs have the potential to exert broad-spectrum antimicrobial activity by targeting diverse and essential proteins in both bacteria and fungi. The application of in silico molecular docking to investigate interactions between nanoparticles and microbial proteins is a valuable approach for understanding their mechanisms of action. For instance, recent studies have successfully employed molecular docking to explain the binding affinities of bioactive agents with nuclear components and stress-response proteins, providing a structural explanation for experimentally reported biological mitigations^78,79^. This methodology has been effectively used in recent studies, such as the work by Daoudi et al. (2024), who employed docking to explore the binding of AgNPs to the SARS-CoV-2 spike protein to explain their antiviral effects. While their study focused on a viral target, it validated the use of computational methods to predict nanoparticle-protein interactions^80^. Building on this concept, our study specifically directs this in silico approach to key fungal proteins to provide, for the first time, a potential molecular explanation for the potent antifungal activity of Ag₂ONPs observed in our experiments.

To provide more insight into the molecular forces, Prime MM-GBSA binding free energy decomposition was performed (Table 8). These findings suggest that van der Waals forces (ΔG_vdw_) have the greatest influence on AgO-NP binding to all four microbial targets, with values ranging from − 3.73 to -5.38 kcal/moL. These values indicate a favorable hydrophobic fit and shape complementarity of the nanoparticles at the protein active site. Changes in hydrogen bonding (ΔG_hbind_) were also observed, particularly for *(A) niger* (negative − 1.20 kcal/moL) and *(B) subtilis* (-1.02 kcal/mol), which provided additional stabilization at the polar interface.

A favorable total binding free energy was observed for *C. neoformans* (+ 2.85 kcal/moL), mainly related to a large desolvation penalty (+ 7.23 kcal/moL). However, the strong negative Van der Waals score (-4.32 kcal/mol) for this strain suggests a strong physical attraction once the nanoparticle reaches the active site In general, analysis of the energy decomposition indicates that non-bonded interactions play a key role in stabilizing the non-bonded interactions.

**Table 7:** Docking score of Ag_2_ONPs with microbial proteins.

Strains	Bacterial Strains			Fungal Strains			
Protein Name	Protein FimH	DNA gyrase subunit B	FtsZ	Laccase-1	Hsp90-like protein	ASPARTIC PROTEINASE (SAP2 GENE PRODUCT)	FDC1 protein
Organism	*E. coli K*-12	*S. aureus*	*B.subtilis*	*C. neoformans*	*T. rubrum*	*C. albicans*	*A. niger*
PDB/Alphafold-ID	4XO8	3U2D	2VAM	AF-Q55P57-F1-v6	AF-F2SVB4-F1-v6	1EAG	4ZA7
Docking Scores							
AgO-NP	-2.4671	-2.50169	-2.43946	-2.43859	-2.45627	-2.43152	-2.44888

**Table 8:** Prime MM-GBSA binding free energy decomposition for AgO NPs docked against bacterial and fungal targets. All energy values are expressed in kcal/moL.

Target Strain		Protein Name	PDB/ID	ΔG_bind_ (Total)	ΔG_vdw_ (Van der Waals)	ΔG_hbind_ (Hydrogen Bond)	ΔG_coulomb_ (Electrostatic)	ΔG_solv−GB_ (Solvation)
Bacterial	*E. coli*	Protein FimH	4XO8	-3.82	-4.40	-0.47	0.00	1.06
	*S. aureus*	DNA gyrase B	3U2D	-3.18	-5.38	-0.01	0.00	2.21
	*B. subtilis*	FtsZ	2VAM	-2.83	-4.44	-1.02	0.00	2.63
Fungal	*T. rubrum*	Hsp90-like	AF-F2SVB4	-3.19	-4.62	-0.09	0.00	1.52
	*A. niger*	FDC1 protein	4ZA7	-2.66	-4.42	-1.20	0.00	2.96
	*C. albicans*	Aspartic Proteinase	1EAG	-2.00	-3.73	-0.13	0.00	1.86
	*C. neoformans*	Laccase-1	AF-Q55P57	2.85*	-4.32	-0.05	0.00	7.23

## Conclusion

This study demonstrated, for the first time, the green synthesis of silver oxide nanoparticles (Ag₂ONPs) using an aqueous flower extract of *N. cataria*. The extract acted as a potent dual-function agent, efficiently mediating the reduction of silver ions and the capping and stabilization of the nanoparticles. Thorough characterization validated the formation of stable, well-crystallized, quasi-spherical Ag_2_ONPs. The synthesized Ag₂ONPs exhibited potent, broad-spectrum antimicrobial activity with high inhibition zones against pathogenic bacteria (a highest of 30 mm against *Bacillus subtilis*) as well as fungi (a maximum of 83 mm against *Trichophyton rubrum*), a finding supported by in silico molecular docking studies predicting strong binding to key microbial proteins. Moreover, the nanoparticles exhibited high hemocompatibility with slight hemolysis. It is important to note that they also had important dose-dependent anticoagulant and thrombolytic effects, highlighting their capacity to regulate blood coagulation. In summary, the flower extract of *N. cataria* is a novel, environmentally friendly, and inexpensive material for producing biocompatible Ag_2_ONPs. The combination of effective antimicrobial performance and promising hemo-modulatory properties makes these green-synthesized nanoparticles potent candidates for various biomedical applications, such as advanced wound dressings, antimicrobial surfaces, and the development of new therapeutic agents for treating thrombotic diseases.

## Supplementary Information

Below is the link to the electronic supplementary material.

Supplementary Material 1

## Data Availability

All the data is contained within the article.
