# Supplementary material for: Eco-friendly synthesis of silver oxide nanoparticles using Nepeta cataria L. (Lamiaceae) flowers extract: a multifaceted study of their antimicrobial and hemocompatible potential
Source: Sci Rep. 2026 May 21;16:23189. doi: 10.1038/s41598-026-53571-8 (PMC13396390; doi:10.1038/s41598-026-53571-8)
Supplement: Supplementary file 1 — Supplementary Material 1 [file 41598_2026_53571_MOESM1_ESM.docx]

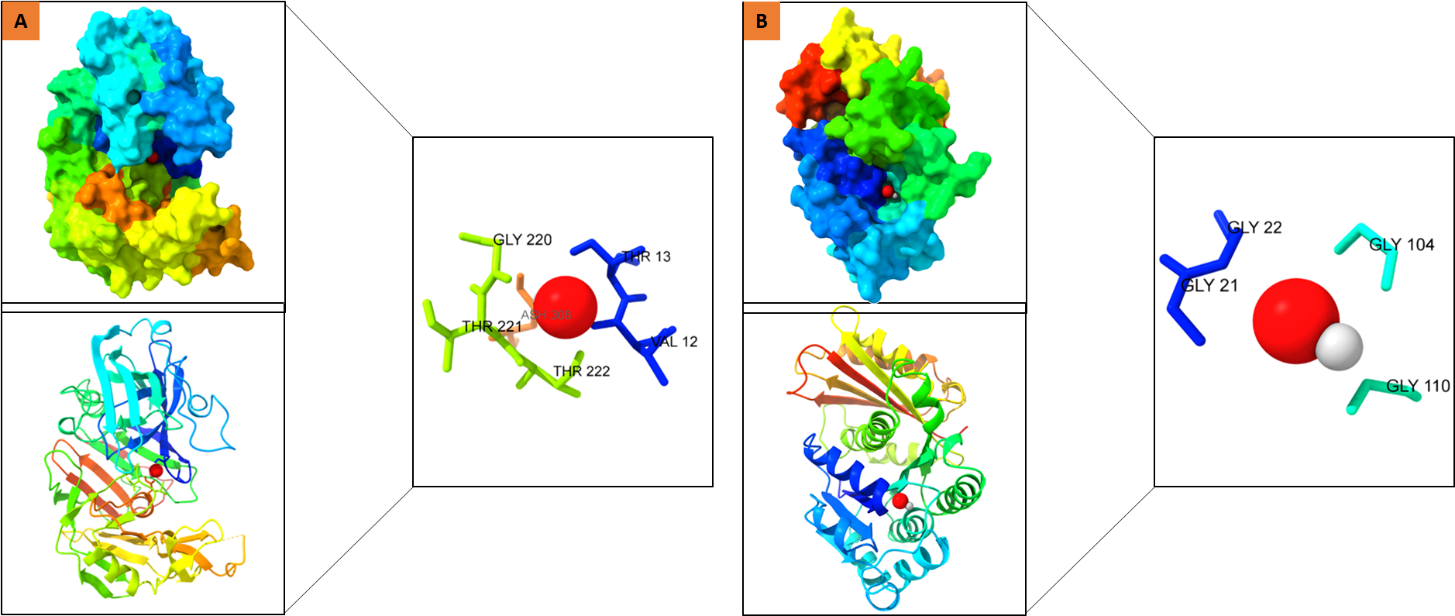


**Figure 1S.** (A) fungal protein, Secreted Aspartic Protease 2 (SAP2) from *Candida albicans*, including its PDB database identifier (1EAG) (B) bacterial protein, Cell division protein FtsZ from *Bacillus subtilis*, including its PDB database identifier (2VAM).


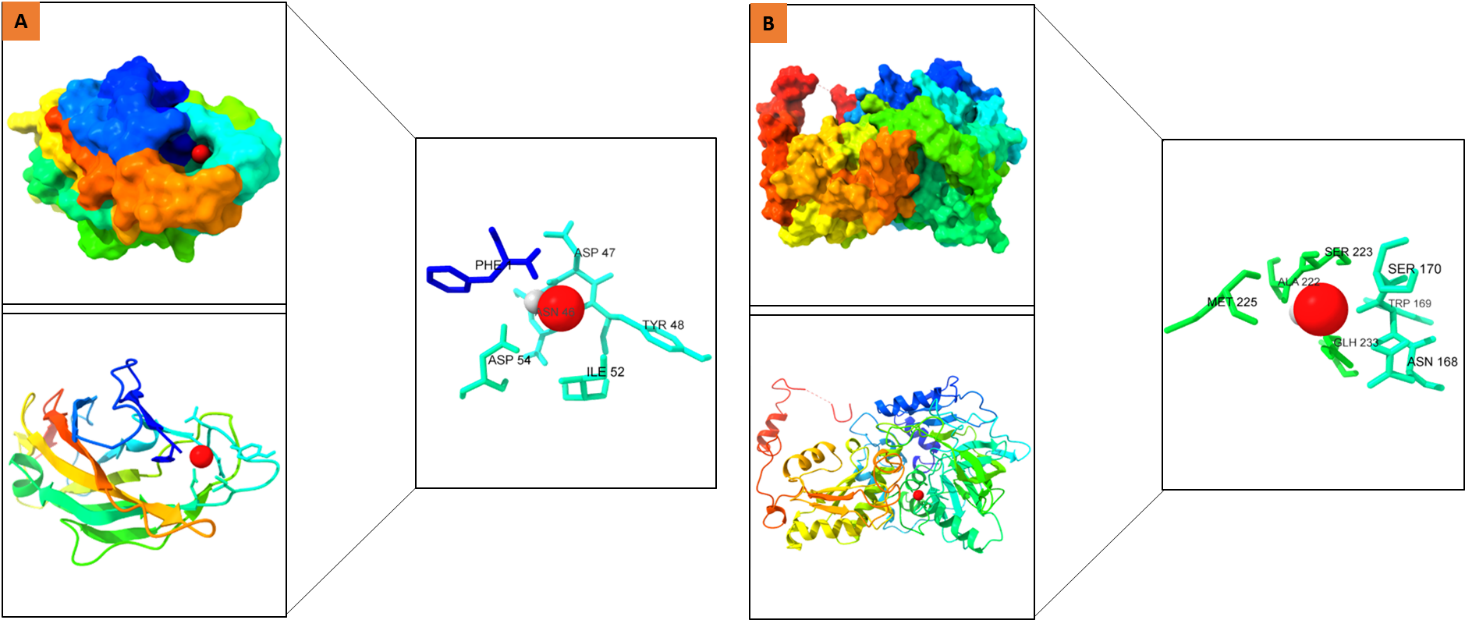


**Figure 2S.** (A) bacterial protein, Type 1 fimbrial adhesin (FimH) from *Escherichia coli* K-12, including its PDB database identifier (4XO8) (B) fungal protein, Ferulic acid decarboxylase 1 (Fdc1) from *Aspergillus niger*, including its PDB database identifier (4ZA7).


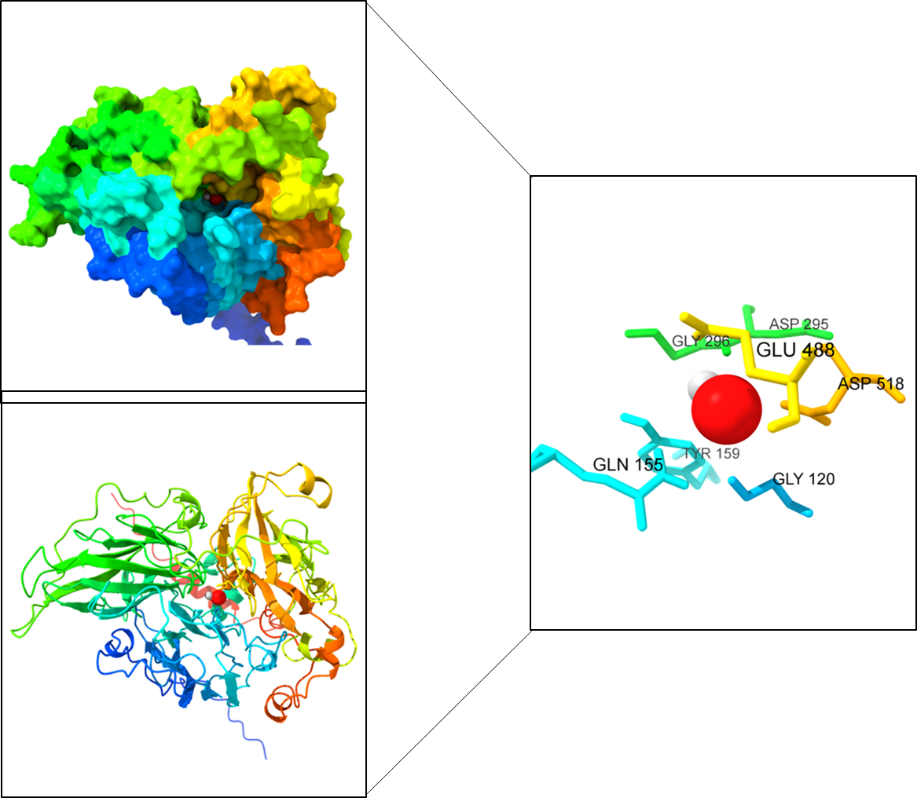


**Figure 3S.** Fungal protein, Laccase-1 (LAC1) from *Cryptococcus neoformans*, including its AlphaFold database identifier (AF-Q55P57-F1-model_v6).

**Table 1S.** Interaction profile of analyzed protein structures. A listing of interacting residues and specific bond types (hydrogen bond, carbon, and metal coordination) associated with each PDB and AlphaFold identifier.

| PDB/Alpha Fold ID’s | Interacting Residues | Conventional H-Bond | Carbon | Metal Donor | Metal Acceptor |
| --- | --- | --- | --- | --- | --- |
| 4XO8 | PHE1, ASP47, TYR48, ASN46, ASP54 | PHE1, ASP47, TYR48, ASN46 |  | PHE1, ASP 47 | ASN46, ASP54 |
| 3U2D | VAL79, THR173 | VAL79, THR173 | THR173 |  | VAL79, THR173 |
| 2VAM | GLY110 |  |  | GLY110 |  |
| AF-Q55P57-F1-v6 | GLY120, ASP518, TYR159, ASP295, GLU488 | GLY120, ASP518 |  |  | TYR159, ASP295, GLU488 |
| AF-F2SVB4-F1-v6 | ASN583 | ASN583 |  | ASN583 |  |
| 1EAG | THR222, VAL12, GLY220, THR13, VAL12, ASH308 | THR222, VAL12, GLY220 | THR13 |  | VAL12, ASH308 |
| 4ZA7 | SER223, SER170, ALA222, MET225, GLH233 | SER223, | SER170 |  | ALA222, MET225, GLH233 |
